# A Membrane‐Targeted Photosensitizer Prevents Drug Resistance and Induces Immune Response in Treating Candidiasis

**DOI:** 10.1002/advs.202207736

**Published:** 2023-10-24

**Authors:** Ming‐Yu Wu, Xiaoyu Xu, Rui Hu, Qingrong Chen, Luojia Chen, Yuncong Yuan, Jie Li, Li Zhou, Shun Feng, Lianrong Wang, Shi Chen, Meijia Gu

**Affiliations:** ^1^ Department of Gastroenterology Ministry of Education Key Laboratory of Combinatorial Biosynthesis and Drug Discovery TaiKang Center for Life and Medical Sciences Zhongnan Hospital of Wuhan University School of Pharmaceutical Sciences Wuhan University Wuhan 430071 China; ^2^ Sichuan Engineering Research Center for Biomimetic Synthesis of Natural Drugs School of Life Science and Engineering Southwest Jiaotong University Chengdu Sichuan 610031 China; ^3^ Department of Respiratory Diseases The Research and Application Center of Precision Medicine The Second Affiliated Hospital of Zhengzhou University Zhengzhou University Zhengzhou 450014 China; ^4^ Department of Medical Intensive Care Unit Maternal and Child Health Hospital of Hubei Province Tongji Medical College Huazhong University of Science and Technology Wuhan Hubei 430070 China

**Keywords:** candidiasis, drug resistance, immune response, photodynamic therapy, photosensitizer

## Abstract

*Candida albicans* (*C. albicans*), a ubiquitous polymorphic fungus in humans, causes different types of candidiasis, including oral candidiasis (OC) and vulvovaginal candidiasis (VVC), which are physically and mentally concerning and financially costly. Thus, developing alternative antifungals that prevent drug resistance and induce immunity to eliminate *Candida* biofilms is crucial. Herein, a novel membrane‐targeted aggregation‐induced emission (AIE) photosensitizer (PS), TBTCP‐QY, is developed for highly efficient photodynamic therapy (PDT) of candidiasis. TBTCP‐QY has a high molar absorption coefficient and an excellent ability to generate ^1^O_2_ and •OH, entering the interior of biofilms due to its high permeability. Furthermore, TBTCP‐QY can efficiently inhibit biofilm formation by suppressing the expression of genes related to the adhesion (*ALS3*, *EAP1*, and *HWP1*), invasion (*SAP1* and *SAP2*), and drug resistance (*MDR1*) of *C. albicans*, which is also advantageous for eliminating potential fungal resistance to treat clinical infectious diseases. TBTCP‐QY‐mediated PDT efficiently targets OC and VVC in vivo in a mouse model, induces immune response, relieves inflammation, and accelerates the healing of mucosal defects to combat infections caused by clinically isolated fluconazole‐resistant strains. Moreover, TBTCP‐QY demonstrates excellent biocompatibility, suggesting its potential applications in the clinical treatment of OC and VVC.

## Graphical abstract

A novel membrane‐targeting AIE photosensitizer, TBTCP‐QY, with excellent biocompatibility, is developed for highly efficient treatment of candidiasis. TBTCP‐QY‐mediated PDT efficiently targets oral candidiasis and vulvovaginal candidiasis in vivo, induces immune response, relieves inflammation, and accelerates wound healing to combat infections caused by clinically isolated fluconazole‐resistant strains, suggesting its potential applications for the treatment of candidiasis.

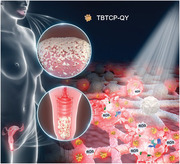

## 1 Introduction

Fungal diseases are a global public health threat, causing more than one billion human infections, and nearly two million deaths annually.^[^ ^1^ ^]^ As one of the most common fungal diseases in the world, candidiasis infections can range from asymptomatic colonization to chronic mucocutaneous and acute systemic candidiasis, such as oral candidiasis (OC), vulvovaginal candidiasis (VVC), and candidemia.^[^ ^2^ ^]^ Although chronic mucocutaneous candidiasis is usually not lethal, it has a serious economic impact, as the total cost in the United States alone was estimated to be approximately two billion dollars in 1998.^[^ ^3^ ^]^ Moreover, candidiasis is the fourth most common case of hospital‐acquired acute systemic infections in the United States, with a mortality rate of up to 50%.^[^ ^4^ ^]^ Currently, conventional antifungal agents, such as those in the polyene (e.g., amphotericin B), azole (e.g., fluconazole, ketoconazole, and miconazole), and echinocandin (e.g., caspofungin) families, remain first‐line drugs and are widely used for treating candidiasis in clinical practice.^[^ ^5^ ^]^ However, fungi respond rapidly to chemical agents.^[^ ^6^ ^]^ The indiscriminate and excessive use of antibiotics results in antimicrobial drug resistance (AMR),^[^ ^7^ ^]^ which greatly exacerbates the difficulties in candidiasis treatment.^[^ ^8^ ^]^ Moreover, the development of new antifungals is challenging, as fungal pathogens use the same eukaryotic machinery as humans, which reduces the number of fungus‐specific targets.^[^ ^9^ ^]^ As resistant infections and “superbugs” are widespread and worldwide,^[^ ^10^ ^]^ the World Health Organization (WHO) has declared AMR to be one of the top 10 global public health threats and has launched strategic framework for collaboration on AMR.^[^ ^11^ ^]^

In October 2022, the WHO published the first‐ever fungal priority pathogen list, identifying 19 fungi with the greatest public health impact and emerging antifungal resistance risk. These pathogens were categorized into three priority groups (Critical, High and Medium), and *Candida* species were designated as the highest priority, “Critical”.^[^ ^12^ ^]^ As listed in the Critical Priority Group, *Candida albicans* (*C. albicans*) is a typical opportunistic pathogen and the most common fungal pathogen in humans, as more than half of all cases of candidemia are caused by *C. albican*s.^[^ ^13^ ^]^ *C. albicans* typically resides in most human mucosal membranes as a lifelong, harmless commensal organism.^[^ ^14^ ^]^ If the balance between the host immune defense system and *C. albicans* is disrupted, *C. albicans* can cause various opportunistic infections at mucocutaneous sites, such as OC and VVC.^[^ ^15^ ^]^ OC is known as the most prevalent fungal disease encountered in dermatology, with a traditionally reported incidence of OC in the general population ranging from 35% to 80%.^[^ ^16^ ^]^ VVC, a widespread infectious disease caused by *Candida* species, affects millions of women every year, with the main symptoms of pruritus vulvae and abnormal vaginal discharge.^[^ ^17^ ^]^ An estimated 75% of women worldwide experience VVC in their lifetime, and 5–8% of them suffer physically and mentally from recurrent VVC (RVVC, defined as >3 episodes per year).^[^ ^18^ ^]^ Several host‐related (e.g., pregnancy) and behavioral (e.g., the use of oral contraceptives) risk factors have been proposed as predisposing factors for VVC.^[^ ^19^ ^]^ Moreover, it has been reported that candidiasis shows much more invasive and destructive characteristics in patients with immunodeficiencies.^[^ ^20^ ^]^ The outer wall of *C. albicans* confers unique properties such as hydrophobicity, adhesion, and diverse components, as well as immunological heterogeneity. These features allow *C. albicans* to thrive in various ever‐changing harsh environments.^[^ ^21^ ^]^ Biofilms, mixtures of fungal cells that are packed in self‐produced, 3D natural physical extracellular polymeric substances (EPSs), are another main cause of AMR, resulting in difficulties in curing candidiasis, such as OC and VVC.^[^ ^22^ ^]^ EPSs offer a favorable microenvironment for the growth of fungi so that they may resist harsh environments and host immune defenses, resulting in strong resistance to antifungals.^[^ ^23^ ^]^ *C. albicans* biofilms are inherently resistant to the majority of known antifungal drugs, making these infections particularly difficult to treat.^[^ ^24^ ^]^ Although many antifungals can penetrate EPS, the cells embedded in the biofilm are often still protected. Antimicrobials require at least some degree of cellular activity to be effective because their mechanism involves the disruption of a microbial process: meanwhile, the polymorphic structure of *Candida* biofilms leads to slow cell metabolism.^[^ ^25^ ^]^ Moreover, a recent study in ***Cell*** defined a biofilm as a protective structure for microorganisms and an aggressive trait that predates different immune cells of the host. The biofilm enfolds immune cells and establishes a high local concentration of a secreted hemolysin to kill immune cells, making the infection more invasive.^[^ ^26^ ^]^ In addition, various mechanisms can lead to acquired resistance in *Candida* species, which is considered another important factor in pathogen drug resistance. The most common mechanism of drug resistance is the induction of efflux pumps by the *MDR* gene to modulate drug exportation.^[^ ^27^ ^]^ The emergence of resistant fungal strains has sparked great efforts to develop new therapies with mechanisms of action that are different from those of conventional antifungal drugs.^[^ ^11b^ ^]^ Developing novel alternatives to conventional antifungals with good penetration ability and biofilm elimination capability to overcome drug resistance in candidiasis and induce immune responses in hosts is highly desirable.^[^ ^22a^ ^]^

In recent years, despite the emerging need for effective antifungal agents, scientists have devoted great efforts to interfering with the growth and metabolism of fungi and preventing the formation or eradiation of fungal biofilms in hopes of countering fungal infections.^[^ ^28^ ^]^ Various novel materials or strategies, including aggregation‐induced emission (AIE) photosensitizers (PSs), nanomaterials, liposomes, microneedles, and graphene, have been reported in antifungal applications.^[^ ^29^ ^]^ Recently, two approaches, both of which integrate multiple therapeutic methods based on a nanozyme platform, were developed and applied for VVC treatment in mice.^[^ ^30^ ^]^ However, the molecular mechanisms of the antifungal activity and biofilm elimination, as well as the clinical potential for related fungal diseases of these novel antifungal therapies in vivo (e.g., efficacy, biocompatibility, administration method, immune response, long/short‐term toxicity), have not been thoroughly investigated. As a promising therapeutic method, photodynamic therapy (PDT) has attracted increasing attention in antibiosis due to its advantages of negligible drug resistance, low systemic toxicity, and minimal side effects.^[^ ^31^ ^]^ PDT utilizes PSs to generate reactive oxygen species (ROS) under light irradiation, which causes oxidative damage to lipids, nucleic acids, or proteins and induces irreversible apoptosis and pathogenic death.^[^ ^32^ ^]^ Since they were first discovered by Tang and coworkers in 2001, AIEgens have gained substantial attention and shown broad prospects in biomedical fields.^[^ ^33^ ^]^ PSs with AIE properties can reduce both nonradiative decay and the energy gap of the intersystem crossing in the aggregate state compared to that in the discrete molecular state, resulting in increased ROS production; thus, AIE PSs have a promising future in the photodynamic elimination of pathogenic microorganisms.^[^ ^34^ ^]^ Although many AIE luminogens (AIEgens) have been synthesized for fungal detection, AIEgens have seldom been developed to combat candidiasis.^[^ ^28^, ^29^, ^30^, ^35^ ^]^ In addition, most PSs generate only ^1^O_2_, making them oxygen dependent. Thus, the anaerobic characteristics of biofilms prevented these PSs from being used in clinical practice.^[^ ^36^ ^]^ As a result, the use of oxygen‐independent PSs is a promising strategy for combating the hypoxic environment of fungal biofilms.^[^ ^37^ ^]^ Moreover, PSs, which can inhibit fungal efflux pumps to overcome drug resistance, have not yet been reported.^[^ ^28^, ^38^ ^]^ Therefore, there is an urgent need to develop more promising antifungals based on oxygen‐independent AIE PSs to treat fungal diseases efficiently.

**Scheme 1:**
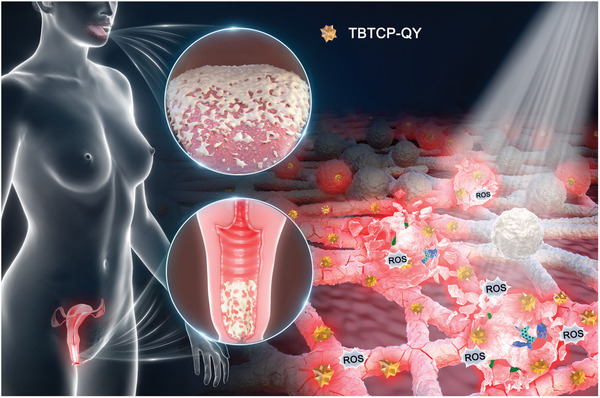
Schematic illustration of the candidiasis treatment with TBTCP‐QY.

Hence, we designed and synthesized a new amphipathic cationic AIE‐active PS, TBTCP‐QY, for effective candidiasis therapy. TBTCP‐QY showed near‐infrared emission, a high quantum yield, a high molar absorption coefficient, and excellent ^1^O_2_ and hydroxyl radical (•OH) generation efficiencies. TBTCP‐QY can selectively bind to the membrane of *C. albicans* through hydrophobic and electrostatic interactions, resulting in an improved photodynamic antifungal effect against planktonic *C. albicans*. Compared with the molecular targets of conventional antifungal drugs, membrane‐targeting represents a more advanced approach, as the membrane structure is less susceptible to change when mutations occur.^[^ ^39^ ^]^ Furthermore, TBTCP‐QY not only prevented biofilm formation and effectively eradicated mature biofilms but also suppressed drug resistance, exhibiting excellent anti‐infective properties in *C. albicans*‐infected mouse models of OC and VVC (**Scheme** **1**). More importantly, the performance of TBTCP‐QY in clinical samples was successfully demonstrated. The highly efficient treatment of candidiasis with TBTCP‐QY‐mediated PDT is expected to open up new avenues for other clinical treatments of fungal infections.

## 2 Results and Discussion

### 2.1 Molecular Design, Synthesis, and Photophysical Properties

**Figure 1:**
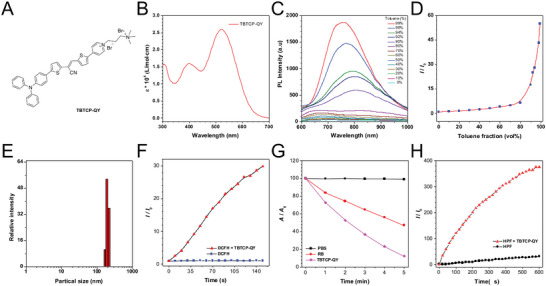
Molecular structure and photophysical properties of TBTCP‐QY. A) Schematic illustration of the structural characteristics of TBTCP‐QY. B) The molar absorption coefficient of TBTCP‐QY in DMSO. C) Photoluminescence spectra of TBTCP‐QY (10 µm) in mixtures of DMSO and PhMe with different PhMe fractions. D) Plot of the relative emission intensity of TBTCP versus PhMe fraction. *I*_0_ and *I* are the peak photoluminescence intensity values of TBTCP‐QY in DMSO and the DMSO/PhMe mixtures, respectively. E) Size distribution of TBTCP‐QY in water. F) Determination of ROS generation by TBTCP‐QY upon white light illumination. Relative changes in fluorescence intensity (*I*/*I*_0_) of DCFH with or without TBTCP‐QY in PBS at 534 nm under white light illumination at different time points. G) Decomposition rates of ABDA in the presence or absence of TBTCP‐QY or RB under light illumination (20 mW cm^−2^), where *A*_0_ and *A* are the initial and final absorbance of ABDA at 378 nm, respectively. H) Determination of •OH generation by TBTCP‐QY upon white light illumination. Relative changes in fluorescence intensity (*I*/*I*_0_) of HPF with or without TBTCP‐QY in PBS at 515 nm under white light illumination at different time points.

Previously, we reported a membrane‐targeted AIE PS that can effectively inactivate human coronavirus and *Streptococcus mutan*s.^[^ ^40^ ^]^ Of note, the cell membranes and cell walls are the fundamental components that isolate the intracellular and extracellular environment of fungi and protect them from external disturbance.^[^ ^41^ ^]^ Current antifungal agents and photosensitive drugs usually have poor membrane penetrability, thus showing poor antibacterial and antifungal effects.^[^ ^39c^ ^]^ Therefore, destroying the cell membrane and cell wall is an important and effective strategy to combat pathogens. Here, an enhanced D–π–A structure was adopted to construct the PS, which can lower the energy gap between the singlet excited state and triplet excited state and is highly beneficial for facilitating intersystem crossing to increase ROS generation efficiency. In our previous work, an AIE‐active plasma membrane‐targeted near‐infrared fluorescent probe, TBTCP, was developed. TBTCP has the inherent attributes of a D–π–A amphiphilic structure and a nonplanar conformation that mimics the structure of phospholipids in the membrane, which is favorable for high ROS generation efficiency and membrane‐targeting. To further increase the permeability of AIEgens to EPSs of biofilms, an additional cationic quaternary ammonium was added to the pyridinium group to obtain TBTCP‐QY (**Figure** **1**A). TBTCP‐QY was synthesized as illustrated in Scheme S1 (Supporting Information), and characterized by ^1^H NMR, ^13^C NMR, and high‐resolution mass spectrometry, as shown in Figures S1–S3 (Supporting Information).

The photophysical properties of TBTCP‐QY were characterized by ultraviolet‐visible spectrophotometry and photoluminescence spectrometry. In dimethyl sulfoxide (DMSO), TBTCP‐QY displayed broad absorption ranging from 300 to 700 nm, with a maximum of approximately 522 nm (Figure 1B). We further studied the AIE properties of TBTCP‐QY in mixed solvents composed of DMSO and different contents of toluene (PhMe). As depicted in Figure 1C, very weak emission was observed in pure DMSO at ≈830 nm with a quantum yield (QY) as low as 0.13%. When the toluene content (*f*_T_) was less than 80%, the fluorescence intensity of TBTCP‐QY did not change with increasing *f*_T_. However, when *f*_T_ surpassed 80%, the maximum emission blueshifted due to the twisted intramolecular charge transfer (TICT) effect and the fluorescence intensity increased dramatically, which was ascribed to the restriction of intramolecular motion (RIM) mechanism induced by aggregation. The fluorescence intensity of TBTCP‐QY in 99% PhMe solution was 55‐fold higher than that in pure DMSO, and the QY increased to 8.74% (Figure 1D). Moreover, the particle size of TBTCP‐QY was measured by dynamic light scattering (DLS) in water, and as shown in Figure 1E, the average size was 143 nm. These data demonstrated the typical AIE properties of TBTCP‐QY.

### 2.2 ROS generation of TBTCP‐QY

A good ROS sensitizing ability is essential for photosensitizers to have a therapeutic effect.^[^ ^42^ ^]^ 2′,7′‐Dichlorodihydrofluorescein diacetate (DCFH) was used here as an indicator to explore ROS generation efficiency. As shown in Figure 1F, upon light irradiation, the fluorescence intensity of DCFH at 525 nm remained constant. In contrast, the fluorescence intensity increased by 30‐fold after light irradiation upon the addition of TBTCP‐QY, demonstrating the high ROS generation efficiency of TBTCP‐QY. Moreover, 9,10‐anthracenediyl‐bis(methylene)dimalonic acid (ABDA) was utilized to study the ^1^O_2_ generation ability. As illustrated in Figure 1G and Figure S4 (Supporting Information), the absorption profile of the ABDA solution hardly changed under white light illumination. However, in the presence of TBTCP‐QY under white light illumination for 5 min, the absorbance of ABDA at 378 nm gradually decreased to 12.4%. For comparison, the widely used PS Rose Bengal (RB) decreased the absorbance of ABDA to only 47.2%. These results showed that TBTCP‐QY is an efficient PS for the generation of ^1^O_2_ and is far superior to RB.

In addition, hydroxyphenyl fluorescein (HPF) was used to investigate the •OH generation ability of TBTCP‐QY for oxygen‐independent PDT. As illustrated in Figure 1H, HPF alone was nearly nonfluorescent, while in the presence of TBTCP‐QY, its fluorescence intensity rapidly increased 376‐fold with increasing irradiation time. Collectively, these results demonstrated that TBTCP‐QY exhibited good ROS production efficiency and could generate both ^1^O_2_ and •OH; thus, TBTCP‐QY holds more potential for antifungal treatment in hypoxic microenvironments, especially for eradicating pathogens in biofilms.

### 2.3 Photodynamic killing of *C. albicans* with TBTCP‐QY

**Figure 2:**
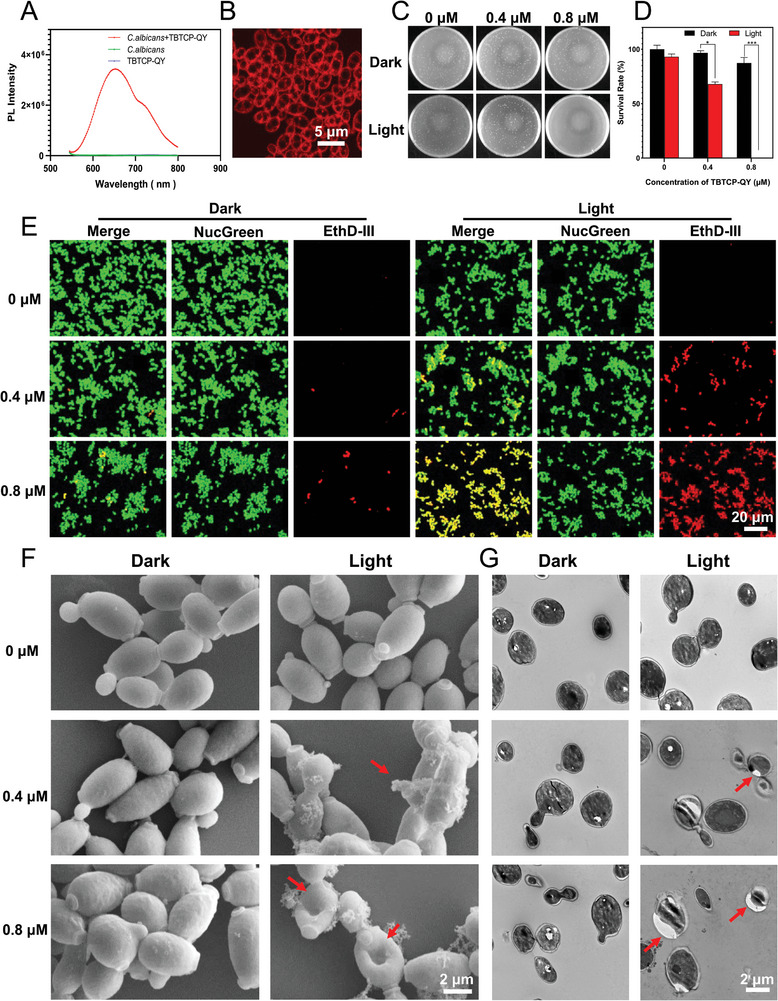
Photodynamic antifungal effect of TBTCP‐QY. A) Photoluminescence spectra of *C. albicans* incubated with or without 5 µm TBTCP‐QY in PBS. Excitation wavelength: 522 nm, bandwidth: 2 nm. B) CLSM images of *C. albicans* incubated with 5 µm TBTCP‐QY. A 561 nm laser and 620–720 nm emission filter were used for imaging. C) Representative images of YPD agar plates employed to quantify *C. albicans* viability. D) *C. albicans* survival rate evaluated by the serial dilution test on YPD agar. *C. albicans* were treated with or without various concentrations of TBTCP‐QY, followed by storage in the dark or under white light irradiation (80 mW cm^−2^) for 15 min. Data are presented as the mean ± SD of at least 3 replicates. E) CLSM images of *C. albicans* after applying a Live & Dead Viability/Cytotoxicity and Assay Kit viability/cytotoxicity assay kit and treatment with various concentrations of TBTCP‐QY under white light irradiation (80 mW cm^−2^) or in the dark for 15 min. A 488 nm laser with a 515–550 nm emission filter (green channel) and a 561 nm laser with a 620–720 nm emission filter (red channel) were used for imaging. F) FESEM and G) TEM morphological images of *C. albicans* incubated with different concentrations of TBTCP‐QY with or without light irradiation for 15 min (80 mW cm^−2^). The red arrows indicate deformed or broken fungal structures. Statistical significance between every two groups was calculated via one‐way ANOVA. *p < 0.05, **p < 0.01, ***p < 0.001, ****p < 0.0001; ns, not significant.

The amphipathicity of TBTCP‐QY is favorable for combining with the cellular plasma membrane or fungal membrane through electrostatic and hydrophobic interactions. First, photoluminescence spectra were analyzed to investigate the interaction between TBTCP‐QY and *C. albicans*. As shown in **Figure** **2**A, TBTCP‐QY emitted very weak emission at ≈730 nm in PBS solution due to the TICT effect. However, once *C. albicans* was added, the photoluminescence intensity dramatically increased with the maximum emission wavelength significantly blueshifted to 650 nm, which was ascribed to the combination of TBTCP‐QY with phospholipids to emit strong fluorescence through RIM.^[^ ^43^ ^]^

Subsequently, fluorescence images of human embryonic kidney 293 (HEK‐293) cells with TBTCP‐QY were acquired using confocal laser scanning microscopy (CLSM). As shown in Figure S5 (Supporting Information), TBTCP‐QY specifically targeted cell surface regions and endowed HEK‐293 cells with high signal‐to‐background fluorescence. Fluorescence images of *C. albicans* with TBTCP‐QY were obtained. As shown in Figure 2B and Figure S6 (Supporting Information), we can clearly observe the outline of *C. albicans*. As illustrated in Figure S7 (Supporting Information), after counterstaining with 4′,6‐diamidino‐2‐phenylindole (DAPI) and TBTCP‐QY, the fungal nuclei were labeled by DAPI with pseudo blue color, while the red emission from TBTCP‐QY lit up the exterior surface. Furthermore, after extending the staining time to 3 h, the membrane of *C. albicans* was still clearly observed (Figure S8, Supporting Information), demonstrating that TBTCP‐QY can precisely and stably anchor onto the fungal membrane.^[^ ^44^ ^]^

We evaluated the PDT effect of TBTCP‐QY on planktonic *C. albicans* by the plate counting method (Figure 2C,D). No antifungal effect was observed without light irradiation, while nearly 95% of *C. albicans* were killed after treatment with 0.4 µm TBTCP‐QY and light irradiation (80 mW cm^−2^) for 15 min. After increasing the TBTCP‐QY concentration to 0.8 µm, all *C. albicans* were killed. Moreover, the PDT effect of TBTCP‐QY on planktonic *C. albicans* was investigated with a Live & Dead viability/cytotoxicity assay kit. NucGreen was used to stain all living and dead fungi with a pseudo‐green color, while EthD‐III was cell membrane impermeable and stained only dead fungi with compromised membranes in a pseudo‐red color. As shown in Figure 2E, under light irradiation (80 mW cm^−2^) alone, all *C. albicans* were alive and showed green fluorescence, demonstrating that this intensity of white light had no killing effect on *C. albicans*. When treated with 0.4 µm TBTCP‐QY in the dark, strong green fluorescence from NucGreen and only dim red fluorescence from EthD‐III were observed (Figure 2E). However, if *C. albicans* were prestained with TBTCP‐QY and then treated with light irradiation, red emission from EthD‐III could be discerned from the fungi, which proved that TBTCP‐QY could kill *C. albicans*. When increasing the concentration of TBTCP‐QY to 0.8 µm, almost all the fungi exhibited bright red fluorescence, demonstrating the good PDT performance of TBTCP‐QY toward *C. albicans*. In addition, a morphological analysis of cell integrity was conducted by electron microscopy (Figure 2F,G). Without light irradiation, *C. albicans* showed intact and smooth surfaces in the absence or presence of TBTCP‐QY. Moreover, after the application of white light, *C. albicans* treated with either 0.4 or 0.8 µm TBTCP‐QY had irregular and shrunken shapes and small cracks as observed by field emission scanning electron microscopy (FESEM) (Figure 2F and Figure S9, Supporting Information). Moreover, transmission electron microscopy (TEM) was utilized to study the changes in the *C. albicans* subcellular structure after treatment. As shown in Figure 2G, the fungi were oval in shape with a dense and homogeneous cytoplasm and an intact cell wall in the dark groups and light‐alone group. However, the cell wall was discontinuous with defects and the cytoplasm was uneven with irregular follicles after treatment with 0.4 µm TBTCP‐QY and light irradiation. The outer surface of the cell had ruptured, and the irregular follicles became even larger after treatment with 0.8 µm TBTCP‐QY (Figure 2G). This suggested that TBTCP‐QY‐mediated PDT disrupted fungal walls and membranes and induced the extrusion of intracellular content. These results unambiguously revealed the direct destructive effect of TBTCP‐QY‐mediated ROS.

### 2.4 Photodynamic Prevention of Biofilm Formation

**Figure 3:**
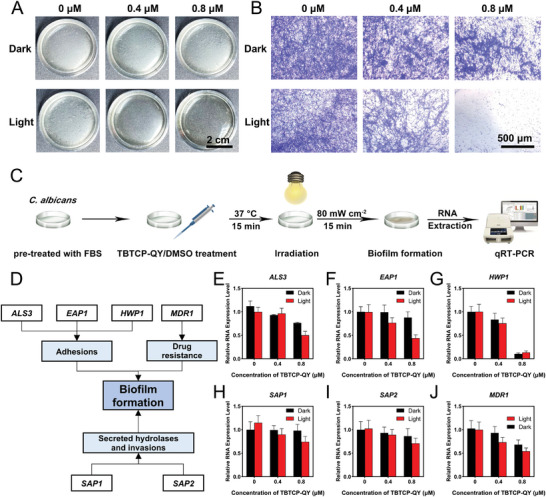
Photodynamic prevention of biofilm formation by TBTCP‐QY. Images of *C. albicans* biofilms formed on A) culture dishes (scale bar: 2 cm) and stained with B) crystal violet after treatment with TBTCP‐QY in the dark or under white light irradiation (80 mW cm^−2^) for 15 min (scale bar: 200 µm). C) The scheme for determining the relative RNA expression of target genes after treatment with and without TBTCP‐QY and light irradiation (80 mW cm^−2^) for 15 min. D) Schematic illustration of the biological functions of the genes *ALS3*, *EAP1*, *HWP1*, *SAP1*, *SAP2*, and *MDR1*. Relative RNA expression levels of E) *ALS3*, F) *EAP1*, G) *HWP1*, H) *SAP1*, I) *SAP2*, and J) *MDR1* from *C. albicans* pretreated with and without TBTCP‐QY and white light irradiation (80 mW cm^−2^) for 15 min. Data are shown as the mean ± SD with three replicates.

*C. albicans* grows and forms biofilms to adapt to a variety of microenvironments, accounting for 70–90% of candidiasis patients.^[^ ^45^ ^]^ Biofilms can protect microorganisms during antimicrobial treatment, leading to persistent infections that seriously endanger human health.^[^ ^23^, ^45^ ^]^ Therefore, effective antifungal agents should not only inhibit the growth of planktonic fungi but also effectively inhibit biofilm formation.^[^ ^46^ ^]^ When *C. albicans* was pretreated with TBTCP‐QY‐mediated PDT, biofilm formation was significantly inhibited, although biofilms could still form when *C. albicans* was treated with TBTCP‐QY alone or light irradiation alone (**Figure** **3**A). The biofilms that formed after different pretreatments were stained with crystal violet and visualized under an inverted microscope.^[^ ^47^ ^]^ As shown in Figure 3B, when applied in combination with light irradiation, TBTCP‐QY significantly suppressed biofilm formation, which was further confirmed by measuring the absorbance at 595 nm quantitatively (Figure S10, Supporting Information).

To gain deeper insight into the underlying mechanisms by which TBTCP‐QY‐mediated PDT inhibits biofilm formation, we determined the expression levels of biofilm‐related genes in *C. albicans* by qRT‐PCR (Figure 3C,D).^[^ ^48^ ^]^ After treatment, total RNA was extracted, and the target genes were quantified with qRT‐PCR by using the *GPD1* gene (encoding Glycerol‐3‐Phosphate Dehydrogenase 1) as an internal reference.^[^ ^49^ ^]^ The primer sequences used are shown in Table S1 (Supporting Information). As shown in Figure 3E–J, TBTCP‐QY downregulated the expression of all these target genes in a dose‐dependent manner. When the concentration of TBTCP‐QY was 0.8 µm, its inhibitory effect in light‐irradiated group was stronger to varying degrees than that in the dark group, suggesting that ROS could suppress the expression of these genes. For comparison, the expression levels of these genes in *C. albicans* treated with light irradiation alone were also measured. The results indicated that light irradiation alone could not suppress the expression of these genes, which is consistent with the antifungal experiments. Agglutinin‐like sequence protein 3, encoded by the *ALS3* gene, plays a crucial role in multiple processes that are necessary for the organism to colonize the host and cause disease, including adherence to host cells and biofilm formation.^[^ ^50^ ^]^ The relative expression of *ALS3* in the 0.8 µm TBTCP‐QY without light irradiation group was ≈80%, whereas the relative expression decreased to 50% with light irradiation (Figure 3E). The *EAP1* gene, which encodes a glycosylphosphatidylinositol‐anchored, glucan‐cross‐linked cell wall protein for adhesion, is required for biofilm formation.^[^ ^51^ ^]^ Incubating *C. albicans* in the dark did not significantly downregulate *EAP1* expression; however, a dose‐dependent downregulation of *EAP1* expression was observed in *C. albicans* treated with TBTCP‐QY and light irradiation (Figure 3F). This trend with TBTCP‐QY‐mediated PDT was similar to that of *ALS3*, suggesting that TBTCP‐QY‐mediated PDT could inhibit *C. albicans* from adhering to host cells and the formation of biofilms. *HWP1* (encoding hyphal wall protein 1) is a crucial gene for biofilm formation and is associated with the adhesion and invasion of *C. albicans*,^[^ ^52^ ^]^ and deletion of *HWP1* leads to the loss of or reduced pathogenicity of the strain.^[^ ^53^ ^]^ Strikingly, the relative expression of *HWP1* decreased to 10% compared with the control after treatment with 0.8 µm TBTCP‐QY, regardless of whether the sample was irradiated (Figure 3G). The secreted aspartic proteases Sap1 and Sap2, encoded by *SAP1* and *SAP2*, respectively, facilitate active penetration by secreting hydrolases to the surface of host cells during biofilm formation. In addition, secreted hydrolases are thought to enhance the efficiency of extracellular nutrient acquisition.^[^ ^4^, ^54^ ^]^ As illustrated in Figure 3H,I, TBTCP‐QY treatment downregulated the expression of these two genes in a dose‐dependent manner. A major mechanism of drug resistance in *C. albicans* is the upregulation of genes encoding efflux pumps that transport toxic compounds out of the cell. *MDR1* encodes the Mdr1 pump, the most important member of the major facilitator superfamily in *Candida* species, and is a frequent mediator of resistance to fluconazole and other toxic compounds in clinical *C. albicans* strains.^[^ ^55^ ^]^ Notably, as shown in Figure 3J, TBTCP‐QY distinctly inhibited the expression of the *MDR1* gene, which might effectively contribute to hindering biofilm barrier function and thereby reducing drug resistance without light irradiation. TBTCP‐QY may interact with components on cell surfaces and regulate the expression of these genes. Collectively, these data show that in addition to directly damaging the structures and biomolecules of *C. albicans* through the ROS it sensitizes, TBTCP‐QY downregulates the expression of genes associated with the adhesion (*ALS3*, *EAP1*, and *HWP1*), invasion (*SAP1* and *SAP2*) and drug resistance (*MDR1*) of *C. albicans*, which could jointly further inhibit the formation of biofilms.

### 2.5 Photodynamic Eradication of Biofilms

Compared with the prevention of biofilm formation, the elimination of mature biofilms is more challenging.^[^ ^56^ ^]^ The poor penetration of conventional antifungal drugs leads to their failure to eradicate biofilms and increases pathogenicity and drug resistance.^[^ ^57^ ^]^ On the other hand, due to the heterogeneous nutrient distribution, fungi in biofilms slow their metabolism to adapt to the severe environment, such as forming spores or persister cells,^[^ ^58^ ^]^ resulting in a drastic reduction in susceptibility to antibiotics.^[^ ^59^ ^]^ Thus, the impact of TBTCP‐QY on mature biofilm eradication was examined.

A biofilm model was constructed by culturing *C. albicans* on the surface of glass slides. TBTCP‐QY (5 µm) was added for incubation with a mature biofilm for 15 min, and fluorescence images were captured by CLSM. As shown in Figure S11, Supporting Information, TBTCP‐QY stained biofilm and the membrane of *C. albicans* hyphae are clearly labeled with red fluorescence. EPSs are electronegative due to their components, such as polysaccharides, proteins, nucleic acids, and lipids.^[^ ^60^ ^]^ Since TBTCP‐QY is amphipathic and has multiple positive charges, it can bind with EPSs and *C. albicans*, thus promoting its penetration into the biofilm and giving it great potential to kill fungi hidden in biofilms effectively. For verification, the ROS‐sensitizing ability of TBTCP‐QY in biofilms was investigated using DCFH as an indicator. DCFH itself is not fluorescent but can produce fluorescence after reaction with ROS. As shown in Figure S12A (Supporting Information), without TBTCP‐QY, no discernable fluorescence was detected from the biofilms. When the biofilm was pretreated with 5 µm TBTCP‐QY in the dark, very weak fluorescence was detected. Once irradiated with light, the bright green fluorescence from DCFH increased dramatically, showing a 17‐fold intensity higher than that in the groups treated with TBTCP‐QY only (Figure S12B, Supporting Information), proving the ROS sensitizing ability of TBTCP‐QY in biofilms.

**Figure 4:**
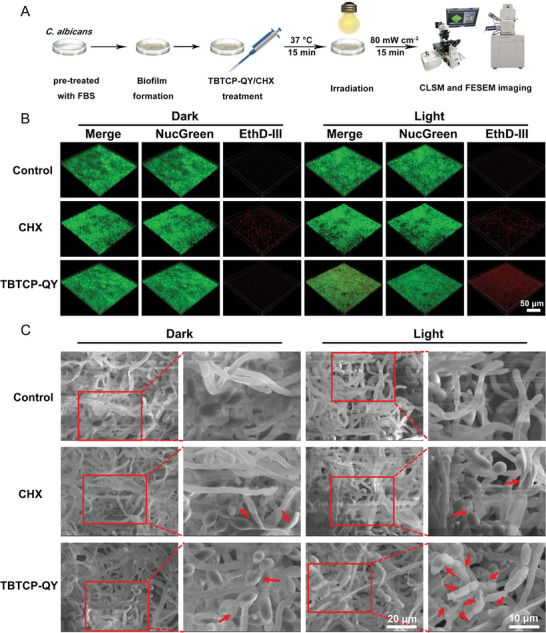
Photodynamic eradication of *C. albicans* biofilms with TBTCP‐QY. A) Scheme for determining the changes in morphology and survival rates of mature *C. albicans* biofilms treated with or without TBTCP‐QY and light irradiation (80 mW cm^−2^) for 15 min. B) Representative 3D images of *C. albicans* biofilms. Thirty‐six‐hour‐old mature biofilms were cocultured with 5 µm TBTCP‐QY or 0.12% CHX in the dark or irradiated with white light for 15 min (80 mW cm^−2^), followed by staining with a Live & Dead Viability/Cytotoxicity assay kit. A 488 nm laser and a 515–550 nm emission filter were employed for the green channel, and a 561 nm laser and a 570–620 nm emission filter were used for the red channel (scale bar: 50 µm). C) Representative FESEM images of *C. albicans* biofilms with different treatments. Mature biofilms were cocultured with 5 µm TBTCP‐QY or 0.12% CHX and then kept in the dark or irradiated with white light for 15 min (80 mW cm^−2^). The red arrows indicate deformed or destroyed fungal and hyphal cell envelopes (scale bar: 20 µm and 10 µm).

Next, the ability of TBTCP‐QY to eradicate biofilms was investigated as reported previously,^[^ ^61^ ^]^ and the data are illustrated in **Figure** **4**A. Biofilms were formed by culturing *C. albicans* on the surface of glass slides. Mature biofilms were incubated with 5 µm TBTCP‐QY in the presence of white light for 15 min; and then rinsed three times with PBS for Live & Dead viability/cytotoxicity assay. Then 3D fluorescence images were recorded by CLSM, as shown in Figure 4B. As a ubiquitous polymorphic species and the most common opportunistic pathogen, *C. albicans* can grow as yeast cells, pseudohyphae, and hyphae.^[^ ^25^, ^62^ ^]^ The heterogeneous structures of the cells in the *C. albicans* biofilms, such as hyphae and spores, were clearly observed in the magnified images (Figure S13, Supporting Information). Without TBTCP‐QY or light irradiation, only the green fluorescence of NucGreen was seen, suggesting that these fungi were viable. When treated with TBTCP‐QY and light irradiation, the strong red fluorescence signal of EthD‐III was detected, indicating that more *C. albicans* were dead. For comparison, the widely used antifungal antibiotic for OC, chlorhexidine (CHX), was used as a positive control at a concentration widely used in clinical practice (0.12%).^[^ ^63^ ^]^ After incubation for 30 min, the killing effect of 0.12% CHX was weak with and without light irradiation, as strong green fluorescence from NucGreen, but the red fluorescence of EthD‐III was dim, demonstrating the poor permeability of CHX, which is consistent with previous reports (Figure 4B).^[^ ^40^, ^64^ ^]^ To quantitatively evaluate the antibiofilm effects, the survival rates of the biofilms were deduced through statistical analysis of the fluorescence intensity and distribution by employing Comstat2. After treatment with 5 µm TBTCP‐QY and light irradiation for 15 min, the survival rate of the fungal biofilm was significantly reduced to 35%, which suggested that TBTCP‐QY‐mediated PDT eradiated fungal biofilms efficiently (Figure S14, Supporting Information). To uncover the mechanism by which TBTCP‐QY‐mediated PDT eradicates fungal biofilms, the morphological changes in the biofilms were evaluated by FESEM. As illustrated in Figure 4C, healthy hyphae with an intact and smooth surface, and homogeneous and round spores were observed in the untreated groups. After treatment with CHX, the hyphae were dim and appeared to have collapsed. After treatment with TBTCP‐QY and light, the hyphae were deformed with apparent holes on the surface and exhibited a collapsed and shriveled shape, while the spores were crumpled. These results clearly demonstrated that TBTCP‐QY could effectively permeate fungal biofilms and kill the fungi embedded in the biofilms with white light irradiation.

### 2.6 In Vivo Photodynamic Antifungal OC Therapy

OC is a common infection caused by *C. albicans* fungi with clinical symptoms that include a sticky, burning sensation in mucous membrane, pain, loss of taste, and the main sign of white curd spots appearing on the oral mucosa. OC usually causes infection in immunocompromised individuals.^[^ ^65^ ^]^ However, due to various virulence factors and environmental adaptabilities of *Candida*, OC can even occur in persons with intact immune systems. The moist and constantly changing conditions within the oral cavity and the presence of saliva, food, and drink offer favorable conditions for the proliferation and adherence of *C. albicans* to the oral mucosa. These conditions also contribute to the short retention time of therapeutics on the mucosal surface and fungal drug resistance, rendering traditional antifungals ineffective.^[^ ^5^ ^]^

**Figure 5:**
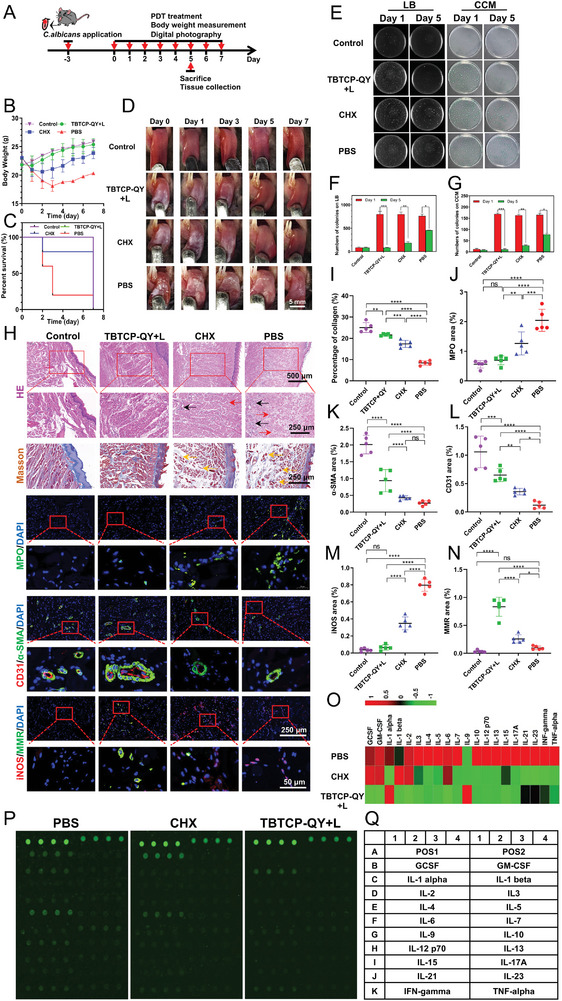
In vivo photodynamic antifungal therapy of OC with TBTCP‐QY. A) Schematic illustration of mucosa defect establishment, treatment and examination. B) Body weight changes, and C) survival curves of mice in different groups. D) Representative images of mice tongues from different treatment groups (TBTCP‐QY+L, CHX and PBS) over 7 days. Mice without *C. albicans* infection was used as the Control group (scale bar: 5 mm). E) Representative plate images and quantitative analysis of samples on F) LB agar and G) CCM agar from the samples collected from the defective regions of mice in different treatment groups (TBTCP‐QY+L, CHX and PBS). Mice without *C. albicans* infection were used as the Control group (scale bar: 250 µm and 500 µm). H) Images of H&E staining, Masson staining (for collagen and muscle fibers) and immunofluorescence staining (MPO staining for neutrophils, CD31/α‐SMA staining for mature blood vessels, iNOS staining for M1 macrophages and MMR staining for M2 macrophages) of the tissues from mice after different treatments on Day 5. The images identify lymphocytes and neutrophils (black arrows), erythrocyte infiltration (red arrows), and disordered structures (yellow arrows). The ratios of I) collagen, J) MPO, K) CD31, L) α‐SMA, M) iNOS, and N) MMR areas in H) (*n* = 5). Data are presented as the mean ± SD. O) Heatmap and P) cytokine array analysis showing significant changes in the expression of Q) multiple proteins from tongues in different treatment groups (TBTCP‐QY+L, CHX, and PBS). Statistical significance between every two groups was calculated via one‐way ANOVA. * *p* < 0.05, ** *p* < 0.01, *** *p* < 0.001, **** *p* < 0.0001; ns, not significant.

Considering its efficient abilities to kill *C. albicans*, prevent the formation of biofilms and eradicate biofilms, the in vivo potential of TBTCP‐QY‐mediated PDT for OC treatment was investigated using 12‐week‐old male C57BL/6 mice (**Figure** **5**A). OC mice were randomly divided into three groups and received the following treatments: 1) PBS, 2) TBTCP‐QY, and light irradiation (TBTCP‐QY+L), and 3) pretreatment with 0.12% CHX. Moreover, healthy mice without OC were used as controls. As shown in Figure 5B, due to the decreased appetites caused by oral mucosal infection, the body weights of the infected mice decreased gradually. The body weights in the PBS and CHX groups continually decreased during the first 3 days, followed by a slow increase over the next 4 days. In the TBTCP‐QY+L treatment group, body weight loss was observed only on the first day and then gradually increased. The mice in the PBS group died due to nutrient intake difficulties resulting from the mucosal lesions during the experiment (Figure 5C). Figure 5D and Figure S15 (Supporting Information) show representative images of OC on Days 0, 1, 3, 5, and 7. Additionally, a white pseudomembrane was clearly observed on the tongues of all OC mice on Day 0. The infection became severe, and the area and thickness of the pseudomembrane increased on Day 1 in both the PBS and CHX groups. The mucosal defect started to recover on Day 3 in the CHX group slowly, and the size of the pseudomembrane decreased gradually. In the PBS group, the pseudomembrane was still clearly observed until Day 7. In contrast, in the TBTCP‐QY+L group, the pseudomembrane disappeared gradually starting on Day 1, and the mucosa had healed by Day 5.

To better differentiate fungi from bacteria, two types of agar plates, LB and *Candida* chromogenic medium (CCM), were used to count colonies in the samples. Bacteria cannot survive and grow in CCM because of the addition of chloramphenicol.^[^ ^66^ ^]^ However, both bacteria and fungi can grow on LB agar. Therefore, the number of colonies on LB agar represented the antibiosis effect for all microorganisms, while the differences in the number of colonies on CCM represent only the antifungal effect. Therapeutic efficacy was assessed by counting the number of colonies on the LB and CCM plates plated with the homogenized wound tissue samples. As shown in Figure 5E–G, on Day 1, many colonies were observed in the PBS, TBTCP‐QY+L, and CHX groups on both LB and CCM plates, demonstrating that the OC mouse model was successfully established. Compared with the PBS and CHX groups, far fewer colonies were detected in the TBTCP‐QY+L group on Day 5, although there were no significant differences on Day 1, between CCM and LB medium. The fungal burdens of the corresponding groups were consistent with the infection images. TBTCP‐QY with light treatment significantly inhibited the growth of fungal and bacterial colonies and showed a much better effect than CHX.

We next performed histological analysis of the wound tissues using hematoxylin and eosin (H&E staining) and Masson trichrome staining methods to observe inflammatory cell infiltration (H&E staining),^[^ ^67^ ^]^ and collagen deposition (Masson staining) in the oral mucosal wounds after 5 days of treatment (Figure 5H).^[^ ^68^ ^]^ Collagen fibers serve as a cellular scaffold in the granulation tissue and promote the formation of new blood vessels and epithelial coverage. The PBS group exhibited fewer collagen fibers and a loose mesh arrangement. Lymphocytes and neutrophils (black arrows), erythrocyte infiltration (red arrows), and disordered structures (yellow arrows) were observed in the CHX and PBS groups, indicating severe mucosal infection. However, the tissues in the TBTCP‐QY+L group showed an intact epidermis with much more collagen and higher myocyte levels (stained red). No significant histopathological differences existed between the control and TBTCP‐QY‐mediated PDT groups, demonstrating that this treatment can effectively remove the fungus and accelerate mucosal healing (Figure 5I).

OC is accompanied by inflammation, which contributes to the injury of oral epithelial cells. During infection, myeloperoxidase (MPO), as an efficient biomarker for wound infection that emerges during the early stage of the infection process, plays a critical role as part of the innate immune system.^[^ ^69^ ^]^ Thus, the expression level of MPO was determined to evaluate the therapeutic effect of TBTCP‐QY in OC. As illustrated in Figure 5J, there was excessive MPO (green) expression in the mucosal tissues in the PBS and CHX groups, revealing the infiltration of neutrophils. In contrast, significantly reduced MPO expression in the TBTCP‐QY+L group suggested that inflammation was significantly suppressed (Figure 5H,J). Neovascularization and vascular maturation are essential to promote mucosa defect healing. To determine whether new vessels had formed, immunostaining for alpha‐smooth muscle actin (α‐SMA, a marker of vascular smooth muscle cells) and platelet endothelial cell adhesion molecule‐1 (CD31, a marker of neovascularization) was carried out.^[^ ^70^ ^]^ As shown in Figure 5H,K,L, the highest expression levels of CD31 and α‐SMA were found in the TBTCP‐QY+L group, demonstrating a superior proangiogenic ability, which allows more nutrients and immune cell trafficking to help mucosal healing.

Macrophages are usually divided into M1 (proinflammatory) and M2 (anti‐inflammatory), which are closely related to inflammatory responses.^[^ ^71^ ^]^ Macrophage polarization is essential for wound healing. Promoting M2 macrophages and inhibiting M1 macrophage polarization is a potential strategy to effectively induce the host immune response to inflammation for spontaneous healing.^[^ ^72^ ^]^ To investigate whether the anti‐inflammatory activity of TBTCP‐QY‐mediated PDT is correlated with polarization of the macrophage phenotype, we utilized immunofluorescence staining to detect the expression of the M1 marker inducible nitric oxide synthase (iNOS) and the M2 marker macrophage mannose receptor (MMR) in wound tissues from tongues after different treatments. As shown in Figure 5H,M, the tissue treated with PBS presented high levels of iNOS, resulting from invasive *C. albicans* infection. Moreover, the CHX group highly expressed iNOS, which might be due to the poor permeability of CHX into *C. albicans* biofilms. However, in the TBTCP‐QY+L group, markedly decreased levels of iNOS (Figure 5M) but increased MMR expression were found compared with the other groups (Figure 5N), which indicated a shift from M1 to M2 macrophages.

Regulating cytokine secretion is essential for maintaining immune system homeostasis, while dysregulation of cytokine production can lead to severe inflammation.^[^ ^73^ ^]^ After the fungal invasion, the host's immune system is activated, and proinflammatory cytokines secreted by M1 macrophages, such as tumor necrosis factor‐α (TNF‐α), interleukin‐1β (IL‐1β), interleukin‐6 (IL‐6), interleukin‐12 (IL‐12), and interleukin‐23 (IL‐23), are produced to recruit leukocytes to kill the pathogen.^[^ ^74^ ^]^ However, the persistence of M1 macrophages can lead to tissue damage. Therefore, M2 macrophages subsequently secrete high levels of interleukin‐10 (IL‐10) and tumor necrosis factor‐β (TNF‐β) to suppress inflammation and contribute to tissue repair and remodeling to maintain homeostasis.^[^ ^71a^ ^]^ To investigate the effect of TBTCP‐QY‐mediated PDT on immune responses, cytokine analysis was carried out.^[^ ^75^ ^]^ The absolute expression levels of different cytokines are shown in Table S2 (Supporting Information). As depicted in Figure 5O–Q, the expression of most proinflammatory cytokines (e.g., IL‐1β, IL‐2, IL‐6, and TNF‐α) in the TBTCP‐QY+L group was significantly lower than that in the other groups, indicating that inflammation was effectively relieved, which is consistent with the immunofluorescence staining results of M2 macrophage polarization. All the above results indicated that PDT with TBTCP‐QY was excellent for inducing an immune response, relieving cytokine storms, and promoting mucosal healing in OC mice.

### 2.7 TBTCP‐QY for VVC Treatment In Vivo

**Figure 6:**
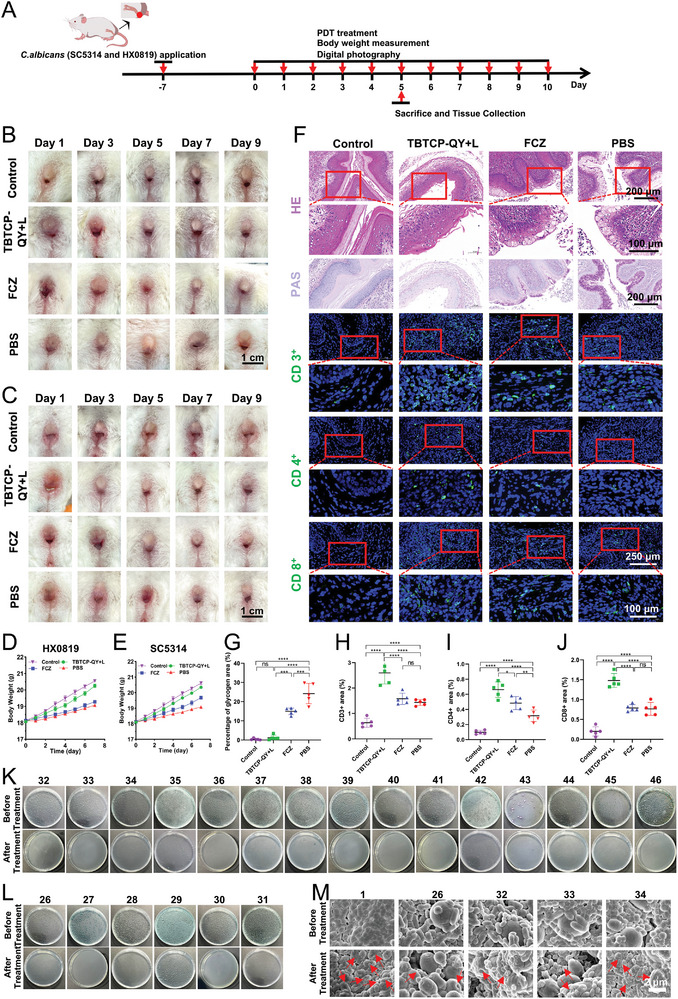
Photodynamic antifungal therapy of VVC in vivo and treatment of clinical samples with TBTCP‐QY. A) Schematic diagram of the time points for vaginitis modeling, treatment, and examination. Representative images of vaginal appearance from mice after different treatments. The VVC model was constructed using B) HX0819 and C) *C. albicans* SC5314 (scale bar: 1 cm). VVC mice were treated with TBTCP‐QY+L, FCZ, and PBS. Mice without *C. albicans* infection was used as the Control group (*n* = 5). Weight changes in mice treated with D) HX0819 and E) *C. albicans* SC5314. F) H&E (scale bar: 100 and 200 µm), PAS (scale bar: 200 µm), and immunofluorescence staining images of CD3, CD4, and CD8 in vaginal tissues from different groups of mice infected with strain HX0819 (scale bar: 100 and 250 µm). Percentages of G) glycogen, H) CD3^+^, I) CD4^+^, and J) CD8^+^ areas in mouse tissue from different groups. K) CCM agar images of clinical samples from patients diagnosed as BV and VVC before and after treatment with TBTCP‐QY+L. L) CCM agar images of VVC clinical samples before and after treatment with TBTCP‐QY+L. M) The morphologies of the samples before and after treatment with TBTCP‐QY+L were observed by FESEM (scale bar: 2 µm). The red arrows indicate deformed or broken bacterial/fungal structures. Data are shown as mean ± SD. Statistical significance between every two groups was calculated via one‐way ANOVA. * *p* < 0.05, ** *p* < 0.01, *** *p* < 0.001, **** *p* < 0.0001; ns, not significant.

Encouraged by its robust therapeutic effect on OC in vivo, we further explored the applications of TBTCP‐QY for VVC.^[^ ^76^ ^]^ To demonstrate the superiority of combating drug‐resistant strains and explore the possibility of clinical application, we screened fluconazole (FCZ)‐resistant strains obtained from clinical patient samples.^[^ ^77^ ^]^ Mouse VVC models were successfully established with the FCZ‐resistant strain HX0819 and *C. albicans* SC5314, and healthy mice without infection were used as the Control group. The overall experimental flow chart is illustrated in **Figure** **6**A. The vagina of each mouse was rinsed with PBS for culture and plate counting on Day 1. As shown in Figure S16, Supporting Information, the agar plates were full of bacterial and fungal colonies, indicating that the mouse VVC models were successfully established. Subsequently, from Day 1 to Day 9, the vaginas of the mice were treated with PBS, FCZ, or TBTCP‐QY and white light (TBTCP‐QY+L). As shown in Figure 6B,C, redness and swelling in the vagina with secretion were observed in all mice infected with HX0819 or SC5314 on Day 1. The mucosal defect did not heal until Day 9 in the PBS group. In the FCZ‐treated groups, the vagina had gradually healed by Day 5 after infection with strain SC5314. In FCZ‐resistant strain HX0819‐infected mice, a similar therapeutic effect was obtained by Day 7. After treatment with TBTCP‐QY+L for 5 days, the inflammation in the wound area was greatly reduced, negligible vaginal secretion was observed, and redness and swelling had almost disappeared in both the SC5314 and HX0819 groups due to the excellent antifungal effect of TBTCP‐QY against both the standard strain and FCZ‐resistant strain of *C. albic*ans (Figure 6B,C). The therapeutic efficacy of TBTCP‐QY was further assessed by counting the colony numbers in the agar plates. As shown in Figure S17, Supporting Information, TBTCP‐QY with light treatment efficiently decreased the colony numbers in both the SC5314 and HX0819 groups on Day 1, and more than 95% of the bacterial and fungal colonies were removed by Day 5. During the whole course of VVC, the body weight of all mice increased gradually (Figure 6D,E).

Furthermore, pathological evaluations were carried out after H&E and periodic acid‐Schiff (PAS) staining to evaluate the therapeutic effects of TBTCP‐QY (Figure 6F–J and Figure S18A,B, Supporting Information).^[^ ^78^ ^]^ The vaginal epithelium maintained a smooth lining morphology without observable irregularities, indicating that TBTCP‐QY‐mediated PDT had remarkable selectivity for eradicating *C. albicans*. In contrast, the keratinized layer of the vaginal mucosal epithelium from the mice treated with PBS was significantly disrupted, and substantial neutrophil infiltration was clearly visible in the interstitium due to fungal infection and inflammatory cells, indicating that inflammation still existed in these mice. Notably, in the model using the SC5314 strain, the keratinized layer of mice in the FCZ group was relatively intact with a few neutrophils in the interstitium. As shown in Figure 6F and Figure S18A (Supporting Information), in the VVC model infected with the FCZ‐resistant strain HX0819, the keratinized layer in the FCZ group appeared disrupted with apparent inflammation. To detect the fungal residue on the infected tissues of the mice on Day 5, PAS staining was used to stain the polysaccharides (Figure 6F,G and Figure S18A,B, Supporting Information). After TBTCP‐QY‐mediated PDT, the number of fungal residues was nearly the same as that from uninfected tissue in both the SC5314‐ and HX0819‐ infected mouse models, while the infection was still obvious in other groups. These results indicated that TBTCP‐QY‐mediated PDT can effectively kill clinical FCZ‐resistant *C. albicans* strains, eradicate their biofilms, and is superior to FCZ.

Although the innate immune system offers a first‐line of defense, T cells are crucial in infectious immunity through their efficient clearance of pathogens (e.g., viruses, fungi, bacteria, and protozoa).^[^ ^79^ ^]^ T lymphocyte subsets are important immune cells in the body that play important roles in specific resistance to pathogens and the regulation of immune system function.^[^ ^80^ ^]^ Pathogen infection is often accompanied by a host response and changes in the ratio of lymphocyte subsets.^[^ ^81^ ^]^ Their cellular function depends on the total number of mature T lymphocytes (CD3^+^ cells) and their subsets (CD4^+^ helper T cells and CD8^+^ suppressor T cells). To clarify the changes in the immune microenvironment after TBTCP‐QY‐mediated PDT in vivo, immune cell levels were measured from the vaginal tissues of VVC mice. As shown in Figure 6F,H–J and Figure S18A,C–E, Supporting Information, due to *C. albicans* infection, immune systems respond to pathogen‐associated molecular patterns and release immune cells, leading to a higher level of T lymphocytes in the PBS group than in the Control group.^[^ ^82^ ^]^ Tissues from the TBTCP‐QY+L, FCZ, and PBS groups revealed higher levels of CD3^+^, CD4^+^, and CD8^+^ T lymphocyte subsets than those from the Control group. To our delight, the levels of CD3^+^, CD4^+^, and CD8^+^ T lymphocytes that are responsible for antifungal immunity were significantly higher in the TBTCP‐QY+L group than in the other groups (Figure 6H–J and Figure S18C–E, Supporting Information). Taken together, these findings of increased levels of T‐cell populations (including CD3^+^, CD4^+^, and CD8^+^ T cells) strongly suggested reshaping of the immune microenvironment by the induction of an immune response to combat fungal infection through TBTCP‐QY‐mediated PDT.

### 2.8 Application of TBTCP‐QY for the Treatment of Clinical Vaginitis Samples

Inspired by the results above, we further evaluated the antifungal properties of TBTCP‐QY in clinical samples from patients with vaginitis. Clinically, vaginitis is divided into VVC, bacterial vaginosis (BV), vaginal trichomoniasis (TV), aerobic vaginitis, and mixed vaginitis. Mixed vaginitis is mediated by at least two types of vaginal pathogens.^[^ ^83^ ^]^ Among the various types of mixed vaginitis, BV+VVC is the most prevalent.^[^ ^83c^ ^]^ The overall experimental flowchart is shown in Figure S19A, Supporting Information. The information collected from the patients with the clinical vaginal secretion samples is shown in Table S3 and Table S4, Supporting Information. According to the clinical diagnosis, all the samples were divided into three groups: BV, BV+VVC (mixed vaginitis), and VVC. After treatment with 0.8 µm TBTCP‐QY and exposure to light for 15 min, the samples were cultured, and the colonies on agar plates were counted. Compared with untreated samples, >99% of the pathogens were killed in all the clinical BV samples (Figure S19B, Supporting Information, samples 1–25), BV+VVC samples (Figure 6K and Figure S19C, Supporting Information, samples 32–46), and VVC samples (Figure 6L, samples 26–31) after TBTCP‐QY‐mediated PDT. Although *C. albicans* causes 75–90% of VVC cases, the frequency of infection by other non‐*albicans* species, such as *Candida glabrata* and *Candida tropicalis*, has increased recently.^[^ ^55^, ^84^ ^]^ In addition, the pathogens in sample 43 were *Candida glabrata* and *Candida tropicalis*, which displayed purple and gray colonies on the agar. More than 99% of the killing rate further indicated the broad application prospects of TBTCP‐QY‐mediated PDT against VVC infection caused by non‐*albicans* species (Figure 6K). Furthermore, the changes in the morphology of BV (sample 1), VVC (sample 26), and BV+VVC (samples 32–34) were observed by FESEM. As shown in Figure 6M, most of the pathogens were deformed and had collapsed with evident holes on the surface after treatment with TBTCP‐QY and white light, while the untreated pathogens maintained regular shapes with a smooth surface and cell walls, indicating the potential of TBTCP‐QY for the clinical treatment of vaginitis.

Considering the diversity of the human vaginal microbial community, we conducted the experiment to test whether TBTCP‐QY could affect the symbiotic microbiomes/species. As shown in Figure S20, Supporting Information, TBTCP‐QY can combat bacteria (20 mW cm^−2^, 30 min), thereby it might affect symbiotic microbiomes/species. However, the TBTCP‐QY‐mediated PDT can efficiently eradicate OC and VVC in vivo, induce immune responses, relieve inflammation, and accelerate wound healing to combat infections caused by clinically isolated fluconazole‐resistant strains. It is invasive and superficial with few side effects. This method might impact the symbiotic microbiomes/species around the lesion but may not involve the whole body. Therefore, compared with the traditional treatment for candidiasis, the TBTCP‐QY‐mediated PDT method is still a promising treatment strategy.

Due to the excellent performance of TBTCP‐QY‐mediated PDT to combat vaginitis in clinical samples, a potential alternative to antifungals for the treatment of vaginitis was investigated. The possibility of TBTCP‐QY‐mediated PDT to induce new drug resistance was investigated using FCZ‐resistant strain HX0819 with 10 cycles of TBTCP‐QY+L treatment.^[^ ^85^ ^]^ As shown in Figure S21 (Supporting Information), TBTCP‐QY‐mediated PDT maintained its antifungal activity after 10 treatment cycles. Moreover, as shown in Figure S22 (Supporting Information), the expression level of *MDR1* hardly increased, and strain HX0819 remained highly susceptible to 0.8 µm TBTCP‐QY‐mediated PDT. This finding further demonstrated that TBTCP‐QY‐mediated PDT did not induce drug resistance, even after 10 cycles of induction at low concentrations. This might be due to several reasons. First, the ROS generated by TBTCP‐QY‐mediated PDT affects various fungal cell components, structures, and metabolic pathways, notably different from conventional antibiotics' mechanisms. Second, TBTCP‐QY has no dark toxicity. Therefore, it is difficult for *C. albicans* to “sense danger” without light irradiation, and all the protective and defense mechanisms against oxidative damage are not active.^[^ ^86^ ^]^

### 2.9 Biocompatibility of TBTCP‐QY

**Figure 7:**
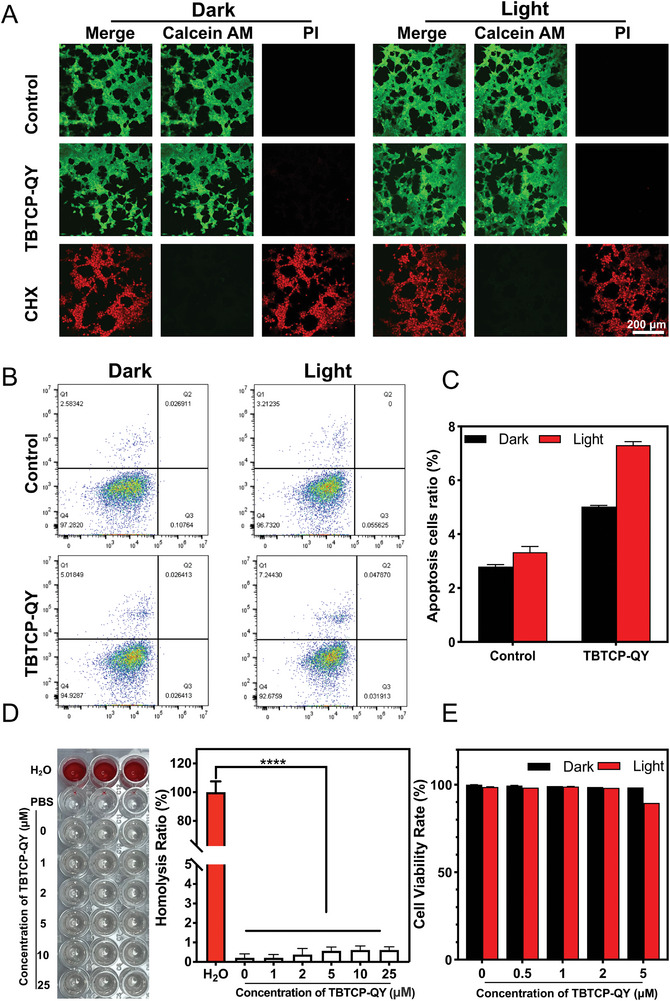
Biocompatibility evaluation of TBTCP‐QY. A) Fluorescence images of HEK‐293 cells pretreated with TBTCP‐QY or CHX (5 h) with or without white light irradiation. The cells were then stained with a Live & Dead Animal Cell Viability/Cytotoxicity Assay Kit (Calcein AM, PI) and observed by CLSM. The green channel used a 488 nm laser and a 515–550 nm emission filter, and the red channel used a 561 nm laser and a 570–620 nm emission filter. B) Flow cytometry analysis and C) quantification of HEK‐293 cells treated without/with 5 µm TBTCP‐QY and white light irradiation (80 mW cm^−2^). D) Evaluation of hemolysis induced by water, PBS and TBTCP‐QY at different concentrations. E) CCK‐8 assay of HEK‐293 cells treated with different concentrations of TBTCP‐QY in the presence and absence of white light irradiation. Data are shown as mean ± SD. Statistical significance between every two groups was calculated via one‐way ANOVA. n = 3, * *p* < 0.05, ** *p* < 0.01, *** *p* < 0.001, **** *p* < 0.0001; ns, not significant.

Encouraged by the outstanding performance of TBTCP‐QY to treat VVC in vitro and in vivo, we further investigated its biocompatibility in vitro and in vivo for potential application in the clinic (**Figure** **7**, Figures S23 and 24, Supporting Information). First, cell viability after treatment was assessed by employing Calcein AM and propidium iodide (PI) as indicators. Calcein AM stain intracellular lactase in living cells with a pseudo‐green color, and PI stain the DNA of dead cells with pseudo‐red color. Thus, dead cells are easily distinguished from live cells. As shown in Figure 7A, after treating HEK‐293 cells with up to 5 µm TBTCP‐QY for 5 h, a neglectable killing effect was observed due to the strong green fluorescence of NucGreen and very weak red fluorescence of PI. However, 0.12% CHX killed almost all the cells stained with PI and exhibited red fluorescence.

We also conducted flow cytometry analysis to gain an intuitive idea of the cytocompatibility of TBTCP‐QY. After incubation with 5 µm TBTCP‐QY and white light irradiation (80 mW cm^−2^) for 15 min, the rate of HEK‐293 cell apoptosis was less than 8%. (Figure 7B,C). Moreover, the potential toxicity of TBTCP‐QY to red blood cells was determined by a hemolysis test. Due to the low osmotic pressure of pure water, the red blood cells in fresh mouse blood rupture and release hemoglobin after incubation in deionized water. At a TBTCP‐QY concentration of 25 μm, the PBS solution remained colorless, which was indicative of a low hemolysis ratio. The absorbance of these solutions was measured at 540 nm (Figure 7D). A CCK‐8 assay was also used to evaluate the cytotoxicity of TBTCP‐QY. HEK‐293 cells were treated with different concentrations of TBTCP‐QY (0, 0.5, 1, 2, and 5 µm) with/without light irradiation for 15 min. As shown in Figure 7E, the cells were still highly viable even after incubation with 5 µm TBTCP‐QY for 5 h. These results demonstrated the excellent biocompatibility of TBTCP‐QY.

To further explore the mechanism underlying the good biocompatibility of TBTCP‐QY, we investigated the interaction between TBTCP‐QY and mammalian cell membranes by zeta potential measurement. As illustrated in Figure S23 and Table S5 (Supporting Information), the zeta potential (ζ) of *C. albicans* significantly increased after incubation with TBTCP‐QY. At the same time, hardly any change was detected for HEK‐293 cells, indicating that TBTCP‐QY had a higher affinity for and binding ability toward *C. albicans* than mammalian cells. The unique structure of the cell wall with the components of an amorphous inner skeletal layer of β(1,3)‐ and β(1,6)‐glucans and β(1,4)‐N‐acetyl glucosamine (chitin) and an outer fibrillar layer endow the plasma membrane of *C. albicans* with more electronegativity than that of mammalian cells.^[^ ^87^ ^]^ Hence, TBTCP‐QY can quickly bind to the surface of *C. albicans* via electrostatic interactions, thus showing selectivity for the membranes of *C. albicans* compared with mammalian cells.

Furthermore, the in vivo biocompatibility of TBTCP‐QY was also investigated. TBTCP‐QY (5 µm) was administered to normal mice for 10 days, and the mice were then sacrificed to obtain the major organs (heart, liver, spleen, lung, kidney), as well as the vagina, uterus, and tongue. H&E staining was then carried out to evaluate the pathological changes. As shown in Figure S24 (Supporting Information), no apparent inflammatory lesions or damage were observed in any of these organs, compared to the Control group. All the above results demonstrated that TBTCP‐QY possessed good biocompatibility, giving TBTCP‐QY great practical application potential.

## 3 Conclusion

In summary, a membrane‐targeted AIE‐active PS, TBTCP‐QY, was designed and developed for the photodynamic elimination of *C. albicans* and to combat candidiasis in vivo. TBTCP‐QY has a high molar absorption coefficient with a broad absorption band covering the whole visible light region, near‐infrared emission, and a high quantum yield. Due to the excellent ^1^O_2_ and •OH generation efficiencies, TBTCP‐QY can be used for the efficient photodynamic elimination of *C. albicans* after selectively binding with and destroying the *C. albicans* membrane. In addition, TBTCP‐QY‐mediated PDT inhibited the formation of biofilms by both producing ROS and downregulating the expression of genes associated with the adhesion (*ALS3*, *EAP1*, and *HWP1*), invasion (*SAP1* and *SAP2*), and drug resistance *(MDR1*) of *C. albicans*. Thanks to the ROS sensitizing ability and amphiphilic attribute of TBTCP‐QY, it also eradicated biofilms, thereby significantly reducing the survival rate of biofilms. EPS removal and fungal envelope deformation were clearly observed by FESEM and TEM, further confirming the impact of TBTCP‐QY‐mediated PDT on biofilms. We also explored the potential of employing TBTCP‐QY‐mediated PDT for treating OC and VVC in vivo. H&E, Masson, PAS, immunofluorescence staining, and cytokine analysis demonstrated that TBTCP‐QY could reinforce anti‐inflammatory responses, increase angiogenesis, relieve cytokine storms, and accelerate mucosal defect healing, showing strong anti‐infection capabilities against both OC and VVC, with excellent biocompatibility. TBTCP‐QY‐mediated PDT was also successfully applied to clinical VVC and BV samples, demonstrating its great potential for clinical candidiasis treatment. TBTCP‐QY is expected to have practical applications for candidiasis treatment in the clinic and may also be generalized to treat other fungal infections.

## 4 Experimental Section

### Materials and General Instruments

All chemical reagents were obtained from J&K Scientific and used without further purification. Dulbecco's Modified Eagle Medium (DMEM, 11 965 092), Phosphate Buffered Saline (PBS, 10 010 031), Penicillin‐Streptomycin (P/S, 15 070 063), heat‐inactivated Fetal Bovine Serum (FBS, 10 100 147), 0.25% trypsin‐EDTA (252 000 056) were purchased from Gibco, Thermo Fisher Scientific, Waltham, USA. Luria‐Bertani medium (LB, HB0128), Nutrient Broth (NB, HB0108), Yeast extract peptone dextrose medium (YPD, HB5193), and *Candida* chromogenic medium (CCM, HB7015) were purchased from Qingdao Hope Bio‐Technology Co., Ltd., Qingdao, China. The Live & Dead Viability/Cytotoxicity Assay Kit (NucGreen/EthD‐III, L6060L), Live & Dead Animal Cells Viability/Cytotoxicity and Assay Kit (Calcein AM/PI, L6037L) were purchased from UElandy, Suzhou, China. DAPI (C0060) was provided from Solarbio, Beijing, China. Mannitol (M813424), Chlorhexidine (CHX, C804720), and Fluconazole (FCZ, F880863) were supplied by Macklin, China. The H&E staining kit (S191003), Masson staining kit (S191006), and PAS staining kit (P0044) were from Pinuofei, Wuhan, China. The bicinchoninic acid (BCA) Protein Assay Kit was from Beyotime Biotechnology, Haimen, China. Milli‐Q water was supplied by the Milli‐Q Plus System (Millipore, MA, USA). An OPPLE LED lamp was employed as the source of white light. The luminescence power was adjusted by tuning the height of the white light source and measured by a Compact Power and Energy Meter Console (PM100D, Thorlabs) together with a Microscope Slide Power Meter Sensor Head (S170C, Thorlabs). Multiska GO microplate spectrophotometer (Thermo Scientific, MA, USA) was employed to measure the photoluminescence spectra in bulk solutions, and a SpectraMax i3x Multi‐Mode Microplate Detection System (Molecular Devices, CA, USA) was used to measure the photoluminescence spectra in 96‐well plates. The Field Emission Scanning Electron Microscope (FESEM, Sigma 300, Zeiss, Germany) and the Transmission Electron Microscope (TEM, HT7800, HITACHI, Japan) were used to collect the morphology images. Fluorescence images were collected with Confocal Laser Scanning Microscope (CLSM, Eclipse Ti‐S, Nikon, Japan) and analyzed by using NIS‐Elements AR software. The zeta potential was measured using a Zetasizer Nano ZS instrument (Malvern, UK). NMR spectra were recorded using a Bruker AMX‐400 or Bruker Avance Neo‐600. Chemical shifts were given in *δ* relative to the internal reference with DMSO‐*d*_6_ as the internal standard. Antibodies used in this study are presented in Table S6 (Supporting Information).

### Synthesis and Characterization of the AIE‐PS

BCN‐PY‐TPA was synthesized according to a previous work.^[^ ^88^ ^]^

BCN‐Py‐TPA (107.5 mg, 0.2 mmol) and 47 mg 3‐bromo‐*N*,*N*,*N*‐trimethylpropan‐1‐aminium bromide (0.18 mmol) in 15 mL acetonitrile were added to a 50 mL round‐bottom flask. The mixture was heated to 100 °C for 24 h. After cooling to room temperature, the solvent was removed under vacuum, and the crude product was purified by silica gel column chromatography with the eluent DCM/MeOH (v/v = 5∶1) to obtain 103.8 mg dark red solid with 65% yield. ^1^H NMR (400 MHz, DMSO‐*d*_6_) *δ*. 9.05 (d, *J* = 4.8 Hz, 2H), 8.49 (d, *J* = 5.6 Hz, 2H), 8.38 (s, 1H), 8.13 (s, 1H), 7.93 (d, *J* = 3.6 Hz, 1H), 7.62 (d, *J* = 7.2 Hz, 2H), 7.51 (dd, *J* = 3.2 Hz, 10 Hz, 2H), 7.36 (t, *J* = 6.0 Hz, 4H), 7.12 (t, *J* = 6.0 Hz, 2H), 7.10 (d, *J* = 6.4 Hz, 4H) 7.00 (d, *J* = 6.4 Hz, 2H), 4.62 (t, *J* = 5.6 Hz, 2H), 3.40‐3.86 (m, 2H), 3.08 (s, 9H), 2.46‐2.41 (m, 2H). ^13^C NMR (150 MHz, DMSO‐*d_6_*) *δ*. 153.0, 152.5, 151.8, 151.1, 150.3, 148.9, 144.7, 141.2, 140.9, 137.8, 136.0, 135.0, 134.7, 131.9, 131.3, 130.0, 129.3, 129.1, 128.1, 127.5, 121.5, 109.9, 67.0, 65.0, 62.1, 29.3. HRMS (ESI): m/z [M – 2Br^−^]^+^ calculated for C_40_H_38_N_4_S_2_: 638.2527; found 638.2563.

### ROS generation efficiency measurement

DCFH was used as the ROS sensor. DCFH (10 µm) was mixed with TBTCP‐QY (2 µm) in PBS in the dark and then exposed to light irradiation for 0–150 s. The fluorescence intensity at 534 nm was monitored by collecting fluorescence spectra with an excitation wavelength of 488 nm.

ABDA was used as the ^1^O_2_ monitoring agent, and RB was employed as the PS standard. ABDA (50 µm) was mixed with TBTCP‐QY or RB (5 µm) in water and exposed to white light illumination (20 mW cm^−2^) for 0–5 min. The absorbance of ABDA at 378 nm was recorded at different illumination times to obtain the decay rate of the photosensitizing process.

The •OH generation measurements were conducted using hydroxyphenyl fluorescein (HPF) as the indicator. A PBS buffer solution containing 5 µm HPF (stock solution: 5 mm in DMF) and TBTCP‐QY (10 µm) was irradiated with white light irradiation for 0–12 min. The fluorescence intensity at 515 nm was recorded with an excitation wavelength of 492 nm.

### Cell and fungi strain culture

*C. albicans* SC5314 were grown in YPD liquid culture medium at 28 °C overnight. The concentration of fungi was determined by measuring the optical density at 600 nm (OD_600_). *C. albicans* SC5314 was kept by the laboratory. The growth of bacteria was inhibited on CCM agar. At the same time, different kinds of *Candida* can grow and demonstrate different colors: *C. albicans* showed a blue‐green color, *Candida tropicalis* showed a gray color, *Candida glabrata* showed a purple color, and other *Candida* showed a white color. Dishes and plates were pre‐treated with FBS overnight for biofilm growth. *C. albicans* were grown at 37 °C in Spider medium (10 g of nutrient broth, 10 g of mannitol, 2 g of K_2_HPO_4_ in 1 L, pH 7.2 after autoclaving).^[^ ^89^ ^]^

*Staphylococcus aureus* (*S. aureus*) ATCC 25 923, Methicillin‐resistant *Staphylococcus aureus* (MRSA) ATCC 43 300, and *Escherichia coli* (*E. coli*) ATCC 25 922 were kept by the laboratory and grown in LB liquid culture medium at 37 °C overnight. The concentration of fungi and bacteria was determined by measuring the OD_600_.

HEK‐293 cells were provided by the China Center for Type Culture Collection. HEK‐293 cells were cultured in DMEM supplemented with 10% heat‐inactivated FBS and 1% antibiotic‐antimycotic (P/S) in a 5% CO_2_ humidified incubator at 37 °C.

### Photoluminescence Measurements

*C. albicans* were cultured at 28 °C until the OD_600_ reached 0.6. After harvesting by centrifuging at 10 000 × *g* for 1 min, the *C. albicans* were washed with PBS 3 times and resuspended in 1 mL PBS containing 5 µm TBTCP‐QY. PBS containing 5 µm TBTCP‐QY and PBS containing *C. albicans* were used as controls. After incubation at 37 °C for 15 min, the mixtures were added to a Corning Costar 96‐well black‐bottom plate (100 µL per well). Photoluminescence spectra were measured by a Molecular Devices SpectraMax i3x multimode microplate detection system. Excitation wavelength: 522 nm, bandwidth: 2 nm.

### Cell Imaging

For cell imaging, HEK‐293 cells were stained with 5 µm TBTCP‐QY for 20 min at 37 °C. After the incubation, the cells were washed with PBS for three times. Confocal imaging was performed using the CLSM. The 561 nm laser and 620–720 nm emission filter were used for TBTCP‐QY. Digital images were captured and processed by NIS‐Elements AR software, as described previously.^[^ ^40^, ^90^ ^]^

### Fungi Imaging

The staining and imaging protocol for fungi was performed according to a previous work.^[^ ^75^ ^]^ In general, *C. albicans* was grown overnight at 28 °C in YPD liquid medium, harvested by centrifugation at 10 000 × *g* for 1 min, and washed twice with PBS. After dilution with PBS to an OD_600_ value of 0.6, the *C. albicans* were cocultured with TBTCP‐QY and DAPI at a final concentration of 5 µm and 10 µm respectively at 37 °C for 15 min and then washed with PBS three times.

To further verify the membrane anchoring stability of TBTCP‐QY, a long‐term imaging experiment was conducted. In general, *C. albicans* was cultured overnight in YPD liquid medium at 28 °C, centrifuged at 10 000 × *g* for 1 min, and washed twice with PBS. After the fungus was suspended with PBS to OD_600_ = 0.6, it was co‐cultured with TBTCP‐QY at a final concentration of 5 µm at 37 °C for different times (5, 10, 20, 60, and 180 min). The fluorescence images were recorded with a CLSM equipped with a 100× oil immersion objective lens and processed using NIS‐Elements AR software. The 561 nm laser and 620–720 nm emission filter were used for TBTCP‐QY, and the 405 nm laser and 450–500 nm emission filter were used for DAPI.

### Biofilm Imaging

For biofilm imaging, mid‐log phase *C. albicans* were harvested and resuspended in Spider medium in confocal dishes with an OD_600_ value of 0.1 and then incubated at 37 °C for 48–72 h. After removing the culture medium and washing twice with PBS, the biofilms were incubated with 1 mL PBS containing 5 µm TBTCP‐QY for 15 min and rinsed three times with PBS. The biofilm images were recorded with CLSM using 60× and 100× oil immersion objective lenses and processed using NIS‐Elements AR software. Excitation wavelength: 561 nm, emission wavelength: 620–720 nm.

### Photodynamic Inactivation of Planktonic Fungi and Bacteria

*C. albicans* were incubated overnight at 28 °C in YPD media, harvested, and diluted with PBS to an OD_600_ value of 0.1 before use. After incubation with different concentrations of TBTCP‐QY (0, 0.4, 0.8 µm) at 37 °C for 15 min, *C. albicans* were washed twice and irradiated with/without white light (80 mW cm^−2^) for 15 min. Fungi were then used for fluorescent staining, plate counting, and FESEM and TEM imaging to determine the viability.

For fluorescent staining, cultures were stained with the Live & Dead Viability/Cytotoxicity Assay Kit following the manufacturer's instructions; dead cells were stained red, while all cells were stained green. *C. albicans* cells were imaged with a CLSM (Eclipse Ti‐S, Nikon). A 488 nm laser and a 515–550 nm emission filter were used for the green channel, while a 561 nm laser and a 570–620 nm emission filter were used for the red channel.

For the plate counting method, 10 µL of *C. albicans* cells diluted with PBS were plated on YPD agar, and the viability of *C. albicans* was assessed by plate counting. Fungal viability was calculated as [colony forming unit (CFU) of the test/CFU of the PBS] 100%. Representative plates were imaged with a Bio‐Rad Universal Hood II‐GelDoc System.

FESEM was adopted to observe the surface morphology of *C. albicans* cells. After centrifugation, the cells were resuspended with glutaraldehyde (2.5% *v*/*v*) at 4 °C for 12 h. After washing with PBS 3 times, the samples were dehydrated in a series of graded ethanol solutions (30%, 50%, 70%, 90%, and 100%) and then successively dried with *tert*‐butanol solution (50%, 75%, 90%, and 100%) for 10 min each. Finally, the cells were dropped on a sterilized silicon wafer, vacuum‐dried until sputter‐coated with gold (20 mA, 60 s), and visualized with FESEM.

TEM was employed to study the internal structural changes of *C. albicans*. Treated cells were fixed with glutaraldehyde (2.5%, v/v) at 4 °C for 12 h and osmic acid (1%, v/v) for 2 h. After washing with PBS three times, the cells were dehydrated with different concentrations of ethanol (30%, 50%, 70%, 90%, and 100%) for 15 min each, treated with pure acetone for 15 min twice, suspended in a graded Spurr812 epoxide resin (30%, 50%, 70%, 100%) for 12 h each, embedded, and left to polymerize for 48 h at 70 °C in an oven. Each sample was cut into thin sections, ≈50–70 nm, using an ultramicrotome and double stained with 2% uranyl acetate and lead citrate. The morphology of *C. albicans* was visualized by a TEM at 100 kV.

*S. aureus*, MRSA, and *E. coli* were cultured in LB medium overnight at 37 °C, harvested, and diluted with PBS to an OD_600_ value of 0.6 prior to usage. The culture medium was removed, and the bacteria were washed twice with PBS and then resuspended in PBS containing TBTCP‐QY at different concentrations. The bacteria were incubated at 37 °C for 15 min. Afterward, TBTCP‐QY‐containing PBS was removed, and the bacteria were washed twice with PBS and then resuspended in PBS. Fifty microliters of each sample was added to a 96‐well microtiter plate and exposed to white light irradiation (20 mW cm^−2^) for 30 min. The bacteria were then subjected to the plate count method and spot plate assay to evaluate their viability.

### Inhibition of Biofilm Formation

*C. albicans* cells were resuspended at OD_600_ = 0.1 with Spider medium. Next, TBTCP‐QY was added to a final concentration ranging from 0 to 0.8 µm in a 96‐well microtiter plate and 40 mm dish, incubated for 15 min at 37 °C, and finally irradiated with/without white light (80 mW cm^−2^) for 15 min. After incubation for 16 h, the planktonic fungi were gently decanted with PBS three times. Digital photographs were then taken of the biofilms in the dishes. Biofilms in the microtiter plate were then fixed with methanol for 15 min and stained with 0.1% crystal violet (CV, 71012314, Sinopharm) for 5 min. The excess dye was removed with PBS and dried, and the biofilms were decolored with 30% acetic acid for 15 min at 37 °C to dissolve evenly. The absorbance was recorded by the Molecular Devices SpectraMax i3x Multi‐Mode Microplate Detection System.

### RNA Isolation and Quantitative Real‐Time PCR (qRT‐PCR)

To further investigate the effects of TBTCP‐QY with/without white light irradiation on biofilm formation, the total RNA of *C. albicans* treated with different concentrations of TBTCP‐QY (0, 0.4, and 0.8 µm) was immediately pretreated with FastPrep−24 5G (MP Biomedicals) and then extracted with TRIzol reagent (15596‐026, Life Technologies). RNA was reverse transcribed using a Hifair II 1st Strand cDNA Synthesis Kit (11119ES60, Yeasen). First, 1 µg of RNA, 1 µL of Oligo (dT)_18_, and 13 µL of H_2_O were denatured at 65 °C for 5 min. After cooling immediately on ice, RNA was reverse transcribed using the following reaction mixture composed of 4 µL of 5× reaction buffer and 2 µL Hifair Enzyme Mix. According to the manufacturer, Amplification conditions were 5 min at 25 °C, followed by 42 °C for 30 min, and deactivation at 85 °C for 5 min. Each 20 µL qRT‐PCR mixture contained 10 µL of Taq Pro Universal SYBR qPCR Master Mix (Q712‐02, Vazyme), 5 µL of cDNA, 0.5 µL of each primer (25 µm), and 4 µL of RNase‐free water. The amplification reaction was performed according to the manufacturer's instructions as follows: 3 min of activation at 95 °C, followed by 40 cycles at 95 °C for 5 s, and a final extension cycle at 60 °C for 30 s. A CFX96TM Real‐Time PCR System (Bio‐Rad, USA) was used for qRT‐PCR, according to the manufacturer's protocol. *C. albicans GPD1* was used to normalize gene expression. The 2^−ΔΔCt^ method was used to determine the relative mRNA expression levels of target genes from three independent replicates of each sample performed in triplicate.

### Measurement of ROS in Biofilms

Mid‐log phase *C. albicans* cells were harvested and resuspended in Spider medium in confocal dishes and 96‐well black plates with an OD_600_ value of 0.1 and then incubated at 37 °C for 48–72 h to form biofilms. Reactive Oxygen Species Assay Kit (S0033, Beyotime) was applied as an indicator of total ROS.^[^ ^23^ ^]^ Commercially available DCFH‐DA was first converted to DCFH by mixing 1 µL of a stock solution of DCFH‐DA in ethanol with 49 µL of 0.01 M NaOH in water and kept at room temperature for 30 min. In brief, the biofilm was washed with PBS, incubated with 100 µL of DCFH (5 µm) for 15 min in the dark at 37 °C, and rewashed with PBS. Then, 100 µL of TBTCP‐QY (5 µm) or PBS was added to the biofilm, incubated for another 15 min at 37 °C, and irradiated with white light (80 mW cm^−2^) for 15 min. The biofilm treated with TBTCP‐QY without irradiation was used as the control. Subsequently, the biofilm was washed with PBS, and fluorescence in confocal dishes was imaged by CLSM. The fluorescence emission intensity of the mixed solutions in 96‐well black plates at 525 nm (excitation wavelength: 488 nm, bandwidth: 2 nm) was recorded using a Molecular Devices SpectraMax i3x multimode microplate detection system.

### Biofilm Eradication Test

The Live & Dead Viability/Cytotoxicity Assay Kit and FESEM imaging were used to study the antifungal effect of TBTCP‐QY treatment on *C. albicans* biofilm eradication. Briefly, a *C. albicans* biofilm was obtained after incubation on 15‐mm confocal dishes for 48–72 h and washed with PBS.^[^ ^91^ ^]^ The biofilm was incubated with 5 µm TBTCP‐QY for 15 min at 37 °C and then irradiated with white light (80 mW cm^−2^) for 15 min. At the same time, other biofilms were incubated with CHX for 30 min, washed with PBS three times, and treated with a Live & Dead viability/cytotoxicity assay kit according to the standard protocol. The biofilm was then imaged by CLSM (Eclipse Ti‐S, Nikon) or prepared for FESEM imaging as described above. The 488 nm laser and 515–550 nm emission filter were employed for the green channel, while the 561 nm laser and 570–620 nm emission filter were used for the red channel.

### OC Animal Model—Construction of the Mouse OC Model

Twelve‐week‐old male C57BL/6 mice were fed drinking water containing 2.5 g L^−1^ tetracycline (T136961, Aladdin) for 5 days to prevent unknown bacterial infection. One day before inoculation, mice were intramuscularly injected with 250 mg kg^−1^ prednisolone to induce immunosuppression.^[^ ^92^ ^]^ Then, the mice were randomly divided into four groups (*n* = 5 in each group). All animal experiments were approved by the Animal Experiment Center of Wuhan University (approval number: WP20220020).

### OC Animal Model—Treatment of OC in Mice

The mice were then divided into four groups as follows:

1) In the PBS group, the tongues of OC mice were washed with 20 µL of PBS every day. 2) In the CHX group, the OC mice were treated with 0.12% CHX every day. 3) In the TBTCP‐QY+L group, the tongues of the OC mice were injected with 20 µL of TBTCP‐QY (5 µm) daily and then illuminated with white light for 15 min. 4) In the Control group, the tongues of mice not infected with *C. albicans* were washed with 20 µL of PBS every day.

Lesions on the lingual mucosa were recorded 72 h after infection. Moreover, to intuitively observe the recovery of the mice from OC, digital photographs were taken to record the mucosa defect healing process continuously.

### OC Animal Model—Bacterial and Fungal Detection

On days 1 and 5, mucosa samples were obtained from all mice with small cotton swabs. One microliter for each sample was diluted with 999 µL of PBS, and the samples were plated on LB and CCM agar and cultured for 12–24 h at 37 °C. Images of the plates were recorded with a Bio‐Rad Universal Hood II‐GelDoc System.

### OC Animal Model—Histological Analysis

Histological analysis of the mucosa defect was carried out on day 5 posttreatment. The mucosa defects were collected and fixed in 4% formaldehyde solution overnight at 4 °C. The pathological sections of wound tissues were analyzed by H&E, Masson, CD31, α‐SMA, iNOS, MMR, and MPO staining. For H&E and Masson staining, the wound tissues were embedded in paraffin, sectioned, and stained with an H&E staining kit (S191003, Pinuofei) and Masson staining kit (S191006, Pinuofei), respectively, following the instructions. The sections were then observed under a microscope (E100, Nikon). To detect the expression of CD31, α‐SMA, iNOS, MMR, and MPO in mucosa defect tissues, frozen tissue sections were permeabilized and blocked with 3% BSA. Then, the sections were incubated with primary antibodies against CD31, α‐SMA, iNOS, MMR, and MPO overnight at 4 °C and then processed with secondary antibodies, while the nuclei were counterstained with DAPI (C0060, Solarbio, 1:500). The stained sections were analyzed by CLSM. Data were quantified by ImageJ software.

### OC Animal Model—Cytokine Antibody Array

Cytokine analysis using Quantibody Mouse Interleukin Array 1 (RayBiotech, Norcross, GA, USA) was carried out to test the expression level of cytokines. After treatment, the tongues of mice of different groups were washed with PBS three times. The samples were centrifuged, and the proteins in the supernatant were quantified by the bicinchoninic acid (BCA) method. Then 100 µL of the original solution was taken from each sample for test, with the procedures according to the manufacturer.

### VVC Animal Model—Screen of Clinical FCZ‐Resistant Strain

Clinical *C. albicans* samples (obtained from the Hubei Maternal and Child Health Hospital) were diluted and plated on CCM overnight at 28 °C to obtain single colonies. Colonies were resuspended in YPD liquid medium containing 32 µg mL^−1^ FCZ and cultured at 28 °C overnight. Among the single colonies, if one of them had the same OD_600_ as the SC5314 strain cultured without FCZ, the single colony was regarded as an FCZ‐resistant strain, named HX0819.

### VVC Animal Model—Construction of the Mouse VVC Model

In this study, 6‐week‐old female BALB/c mice were used to conduct VVC models. Then the mice were randomly divided into different groups (*n* = 5 in each group). All animal experiments were carried out as approved by the Animal Experiment Center of Wuhan University (No. WP20220020). Two VVC models were established: one infected with the standard strain SC5314 and another infected with the clinical‐resistant strain HX0819. SC5314 and HX0819 were cultured at 28 °C overnight to an OD_600_ of 0.25. A 20 µL portion of *C. albicans* suspension was injected into the vagina of the mice, and the mice were kept upside down for 5 min.

### VVC Animal Model—Treatment of VVC in Mice

1) In the PBS group, the vaginas of the VVC mice were washed with 20 µL PBS every day. 2) In the FCZ group, the VVC mice were treated with 0.3 mg of FCZ orally every day. 3) In the TBTCP‐QY+L group, 20 µL of TBTCP‐QY (5 µm) was injected into the vaginas of the VVC mice daily, the mice were inverted for 15 min, and then white light illumination was applied for 15 min. 4) In the Control group, the vaginas of mice not infected were washed with 20 µL of PBS every day. The shape and color of each vagina were recorded 72 h after infection. Moreover, to intuitively observe the recovery of the mice from VVC, digital photographs were taken to record the mucosa defect healing process continuously.

### VVC Animal Model—Bacterial and Fungal Detection

On days 0 and 5, the bacteria and fungi in the vaginal washes were collected by flushing the vaginas with 10 µL of sterile PBS, followed by diluting 1 µL of sample with 999 µL of PBS. The samples were plated on LB and CCM agar and cultured for 12–24 h at 37 °C. Mixtures of the vaginal washes from day 0 were plated on agar to determine whether the VVC models were successfully constructed. Plate images were recorded with a Bio‐Rad Universal Hood II‐GelDoc System.

### VVC Animal Model—Histological Analysis

Mice were sacrificed after 5 days of treatment, and their vaginal tissues were collected and fixed in 4% formaldehyde solution overnight at 4 °C. For H&E and Masson staining, the wound tissues were embedded in paraffin, sectioned, and stained the reagents of with an H&E staining kit (S191003, Pinuofei) and a PAS staining kit (S191008, Pinuofei), respectively, following the instructions. The sections were then observed under a microscope (E100, Nikon). To detect the expression of CD3, CD4, and CD8 in wound tissues, frozen tissue sections were permeabilized and blocked with 3% BSA. Then, the sections were incubated with primary antibodies against CD3, CD4, and CD8 overnight at 4 °C and processed with secondary antibodies. Moreover, the nuclei were counterstained with DAPI (C0060, Solarbio, 1:500). The stained sections were imaged by CLSM. Data were quantified by ImageJ software.

### Clinical Sample Collection and Treatment

Clinical samples were obtained from the Hubei Maternal and Child Health Hospital. The Hubei Maternal and Child Health Hospital approved this study (approval number: 2022IEC025 01/04/2022), and informed consent was obtained from the patients before samples were collected. The fresh samples were collected and immediately stored in liquid LB broth at 4 °C. The samples were diluted with PBS and inoculated on agar plates (CCM for VVC samples, YPD for bacterial vaginitis, and mixed vaginitis samples). TBTCP‐QY (0.8 µm) was added, incubated at 37 °C for 15 min, and irradiated with white light (80 mW cm^−2^) for 15 min. Plate images were recorded with a Bio‐Rad Universal Hood II‐GelDoc System.

### Antimicrobial Photodynamic Inactivation Resistance Testing

To investigate the development of resistance of TBTCP‐QY to antimicrobial photodynamic inactivation (PDI) treatment, 10 cycles of PDI were carried out. The FCZ‐resistant *C. albicans* HX0819 was incubated overnight at 28 °C in YPD media, harvested, and diluted with PBS containing 0.3 µm TBTCP‐QY to an OD_600_ value of 0.1 before use. Each cycle had a total irradiation time of 15 min, and after plating the surviving fungi from the previous PDI cycle on YPD agar and incubating for 18 h at 28 °C, single colonies were isolated, and a new set of fungal cultures was prepared and illuminated. PDI treatments were repeated under similar conditions. After treatment of every cycle, samples were used for fluorescent staining. Three independent experiments were carried out. After ten cycles of PDI, the cells were repeatedly incubated overnight and diluted with PBS containing 0.8 µm to an OD_600_ value of 0.1. Then, the samples were irradiated with white light for 15 min (80 mW cm^−2^) for fluorescent staining and qRT‐PCR. For fluorescent staining, cultures were stained with the Live & Dead Viability/Cytotoxicity Assay Kit (UElandy) and imaged with CLSM. A 488 nm laser and a 515–550 nm emission filter were used for the green channel, while a 561 nm laser and a 570–620 nm emission filter were used for the red channel. To examine the effect of PDI treatment on the expression of *MDR1*, untreated samples, and cells after ten cycles of PDI were incubated overnight at 28 °C. The isolation of RNA and the process of qRT‐PCR were conducted as mentioned above (section RNA Isolation and Quantitative Real‐Time PCR (qRT‐PCR)).

### Biocompatibility Evaluation

Flow cytometry and CCK‐8 assays were conducted to explore the cytotoxicity of TBTCP‐QY, and hemolysis tests were carried out to evaluate biological compatibility.

Cell viability was determined by the standard WST‐8 (2‐(2‐methoxy‐4‐nitrophenyl)−3‐(4‐nitrophenyl)−5‐(2,4‐disulfophenyl)−2H‐tetrazolium, monosodium salt) Cell Counting Kit‐8 assay (CCK‐8, C0040, Beyotime). HEK‐293 cells were seeded at a density of 5 × 10^3^ cells per well in 96‐well microplates with 100 µL of culture medium and cultured overnight to reach 70–80% confluence. Then, the medium was replaced with 100 µL of fresh medium containing different concentrations of TBTCP‐QY (0, 0.5, 1, 2, and 5 µm) and incubated for 10 min at 37 °C. After irradiation under white light for 15 min (80 mW cm^−2^), the samples were incubated at 37 °C for 5 h. DMSO was used as a vehicle control. Then, 10 µL of 12 mM CCK‐8 stock solution mixed with 90 µL of PBS was added to each well for an additional 2.5 h of incubation. The absorbance was measured at 450 nm using a SpectraMax M2 microplate reader (Molecular Devices). Cell viability (%) was calculated as follows: (OD_450_ test/OD_450_ control) × 100%.

For the hemolysis test, fresh mouse blood was centrifuged at 5000 rpm for 10 min to remove the plasma and obtain red blood cells. Red blood cells were then resuspended in PBS (20%, v/v). A mixture of 50 µL cell suspension and 150 µL PBS solution containing different concentrations of TBTCP‐QY was incubated for 30 min at 37 °C. After centrifugation for 10 min at 2000 rpm, the OD_540_ was determined with a microplate reader. The absorbance of different concentrations of TBTCP‐QY was used as the negative control, while the absorbance of sterile water was used as the positive control. The images were captured by a digital camera. The hemolysis ratio (%) was calculated as follows:

(1) $$\mathrm{Hemolysis}\;\%\;=\frac{\mathrm{sample}\;\mathrm{absorbance}\;-\;\mathrm{negative}\;\mathrm{control}}{\mathrm{positive}\;\mathrm{control}\;-\;\mathrm{negative}\;\mathrm{control}}\;\times 100\%$$

CLSM was adopted to observe the viability of HEK‐293 cells. HEK‐293 cells were treated in the same manner as those used for sample preparation for CCK‐8 assay and stained with the Live & Dead Animal Cell Viability/Cytotoxicity Assay Kit (Calcein AM, PI) following the instructions. Dead cells were stained red, while live cells were stained green. Cells were imaged by CLSM, with a 488 nm laser and a 515–550 nm emission filter for the green channel, a 561 nm laser, and a 570–620 nm emission filter for the red channel.

An Annexin V‐FITC/PI Apoptosis Detection Kit (Vazyme) was used to investigate the apoptosis rate. HEK‐293 cells were seeded into six‐well plates and cultured overnight. Then, the medium was replaced with 1 mL of fresh medium containing 5 µm TBTCP‐QY and incubated for 10 min at 37  °C. DMSO was used as a vehicle control. After irradiation under white light for 15 min (80 mW cm^−2^), the cells were digested by trypsin without EDTA and centrifuged at 1000 rpm for 5 min to discard the supernatant solution. The collected cells were resuspended in PBS and counted. Approximately 5 × 10^5^ to 1 × 10^6^ resuspended cells were centrifuged at 1000 × *g* for 5 min. After discarding the supernatant solution, 195 µL Annexin V‐FITC binding solution, 5 µL Annexin V‐FITC, and 10 µL of PI staining solution were added sequentially and mixed gently. The cells were incubated at room temperature for 10–20 min and then tested with a CytoFLEX cytometer (Beckman‐Coulter). For FITC: λ_ex_ = 375 nm, λ_em_ = 400–500 nm. For PI: λ_ex_ = 488 nm, λ_em_ = 590–630 nm.

After 10 days of TBTCP‐QY (5 µm) treatment administered to mice, the heart, liver, spleen, lung, kidney, vagina, uterus, and tongue tissues of mice were obtained for HE staining to investigate biocompatibility, *n* = 5.

### Zeta Potential Measurements

Zeta potential measurements were taken when *C. albicans* reached the log phase of growth. *C. albicans* were treated with 5 µµ TBTCP‐QY for 10 min. HEK‐293 cells were used for zeta potential measurements after cultures reached 70% confluency. Trypsinization of the cells was performed very quickly to minimize changes to the plasma membrane. After trypsinization, the cells were resuspended in 1 mL of PBS and treated with 5 µm TBTCP‐QY for 10 min. The zeta potential was measured before and after treatment with TBTCP‐QY at 25  °C from a minimum of 3 samples (maximum 100 runs each) using a Zetasizer Nano ZS instrument (Malvern, UK) and disposable zeta potential cells with gold‐coated platinum electrodes (DTS1070, Malvern, UK). The cuvettes were always rinsed twice with PBS between each measurement.

### Statistical Analysis

All experiments were performed with at least three replicates. All data were statistically analyzed using GraphPad Prism 8 statistical software. The apoptosis data were statistically analyzed by FlowJo V10. The 2D images and fluorescence intensities were processed or analyzed by ImageJ. The 3D images were processed using NIS‐Elements AR software (Nikon) and Comstat2. All data were presented as the mean ± standard deviations (SD) except where indicated otherwise. One‐way analysis of variance (ANOVA) followed by Tukey's post hoc test was used for comparisons among multiple groups. For comparison between different groups, significant differences with untreated samples were calculated as controls (*p < 0.05; **p < 0.01; ***p < 0.001; ****p < 0.0001; ns, not significance).

## Conflict of Interest

The authors declare no conflict of interest.

## Supporting information

Supporting Information

## Data Availability

The data that support the findings of this study are available from the corresponding author upon reasonable request.
