# Supplementary material for: A Membrane‐Targeted Photosensitizer Prevents Drug Resistance and Induces Immune Response in Treating Candidiasis
Source: Adv Sci (Weinh). 2023 Oct 24;10(35):2207736. doi: 10.1002/advs.202207736 (PMC10724446; doi:10.1002/advs.202207736)
Supplement: Supplementary file 1 — Supporting Information [file ADVS-10-2207736-s001.pdf]

ADVANCED SCIENCE
Open Access

# Supporting Information

for *Adv. Sci.*, DOI 10.1002/advs.202207736

A Membrane-Targeted Photosensitizer Prevents Drug Resistance and Induces Immune Response in Treating Candidiasis

*Ming-Yu Wu, Xiaoyu Xu, Rui Hu, Qingrong Chen, Luojia Chen, Yuncong Yuan, Jie Li, Li Zhou, Shun Feng, Lianrong Wang*, Shi Chen* and Meijia Gu**

# Supporting Information

## A Membrane-Targeted Photosensitizer Prevents Drug Resistance and Induces Immune Response in Treating Candidiasis

*Ming-Yu Wu[#], Xiaoyu Xu[#], Rui Hu[#], Qingrong Chen, Luojia Chen, Yuncong Yuan, Jie Li, Li Zhou, Shun Feng, Lianrong Wang[*], Shi Chen[*] and Meijia Gu[*]*

Dr. M-Y. Wu, Ms. X. Xu, Ms. R. Hu, Ms. Q. Chen, Ms. L. Chen, Mr. Y. Yuan, Dr. L. Zhou, Dr. Z. Li, Prof. L. Wang, Prof. S. Chen and Prof. M. Gu

Department of Gastroenterology, Ministry of Education Key Laboratory of Combinatorial Biosynthesis and Drug Discovery, TaiKang Center for Life and Medical Sciences, Zhongnan Hospital of Wuhan University, School of Pharmaceutical Sciences, Wuhan University, Wuhan 430071, China

Dr. M.-Y. Wu and Prof. S. Feng

Sichuan Engineering Research Center for Biomimetic Synthesis of Natural Drugs, School of Life Science and Engineering, Southwest Jiaotong University, Chengdu, Sichuan, 610031, China

Ms. R. Hu, Prof. L. Wang and Prof. S. Chen

Department of Respiratory Diseases, The Research and Application Center of Precision Medicine, The Second Affiliated Hospital of Zhengzhou University, Zhengzhou University, Zhengzhou 450014, China

Dr. J. Li

Department of Medical Intensive Care Unit, Maternal and Child Health Hospital of Hubei Province, Tongji Medical College, Huazhong University of Science and Technology, Wuhan, 430070, Hubei, China

E-mail: lianrong@whu.edu.cn, shichen@whu.edu.cn, mjgu@whu.edu.cn

[#] M-Y. W., X. X. and R. H. contributed equally to this work.

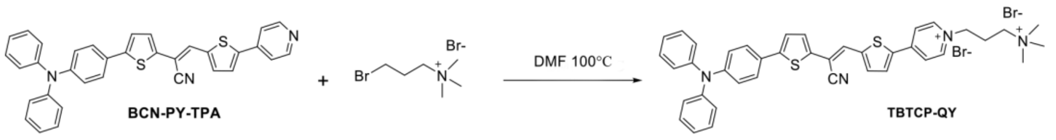


**Scheme S1.** Synthetic route to TBTCP-QY.

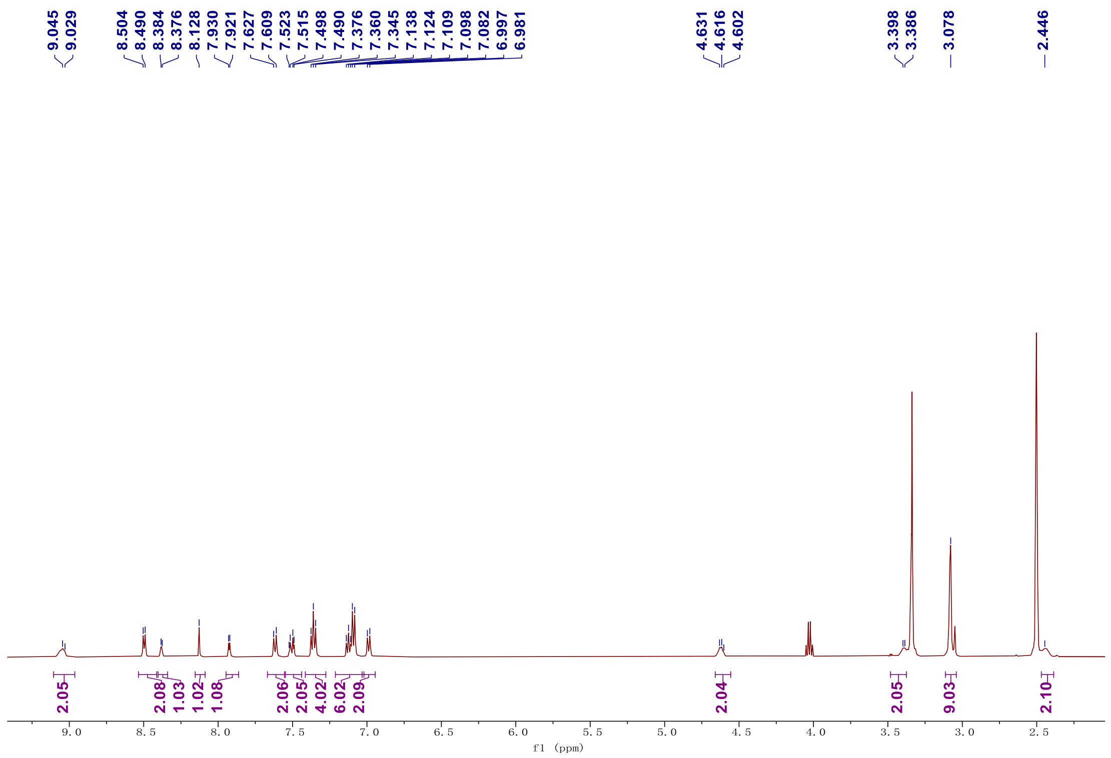

**Figure S1.** $^{1}$H NMR spectrum of TBTCP-QY in DMSO-$d_6$.

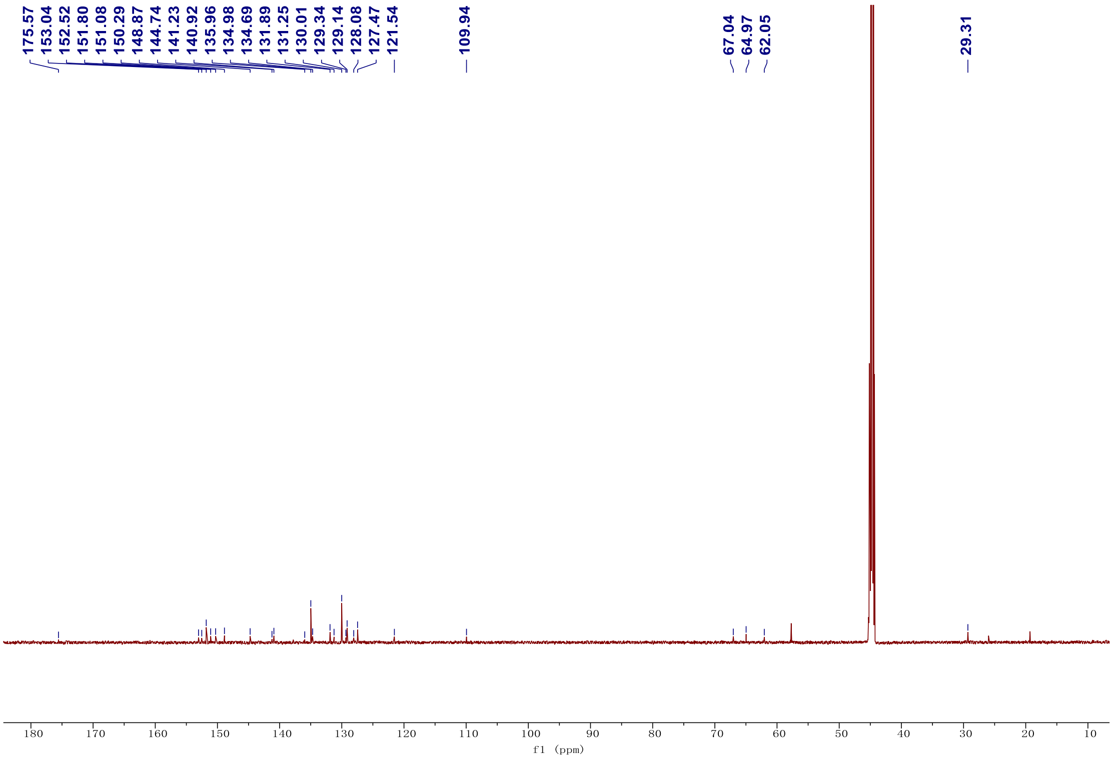


**Figure S2.** $^{13}C$ NMR spectrum of TBTCP-QY in DMSO-$d_6$.

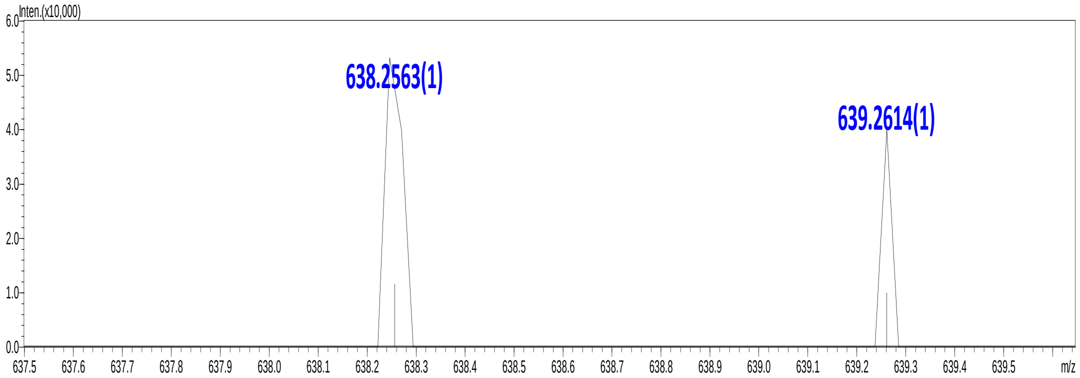


**Figure S3.** HRMS spectrum of TBTCP-QY.

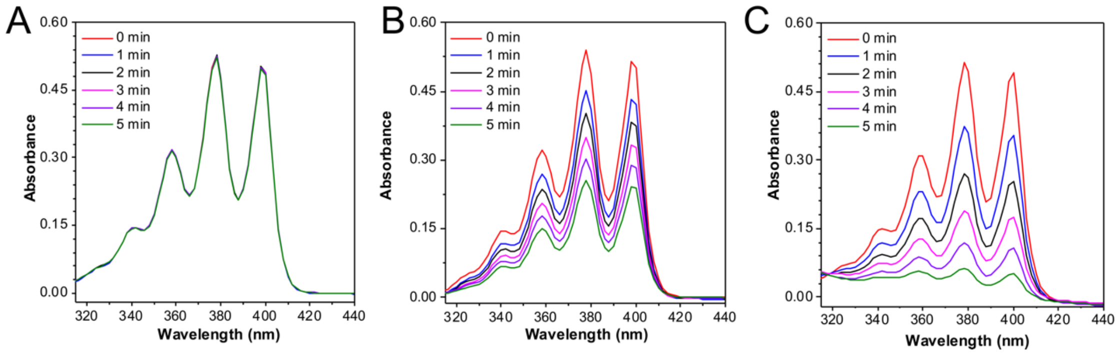


**Figure S4.** Evaluation of TBTCP-QY-sensitized $^1O_2$ production with ABDA. A) Absorption spectra of ABDA (50 μM) in PBS or in the presence of B) 5 μM Rose Bengal and C) TBTCP-QY under white light (20 mW $cm^{-2}$) irradiation.

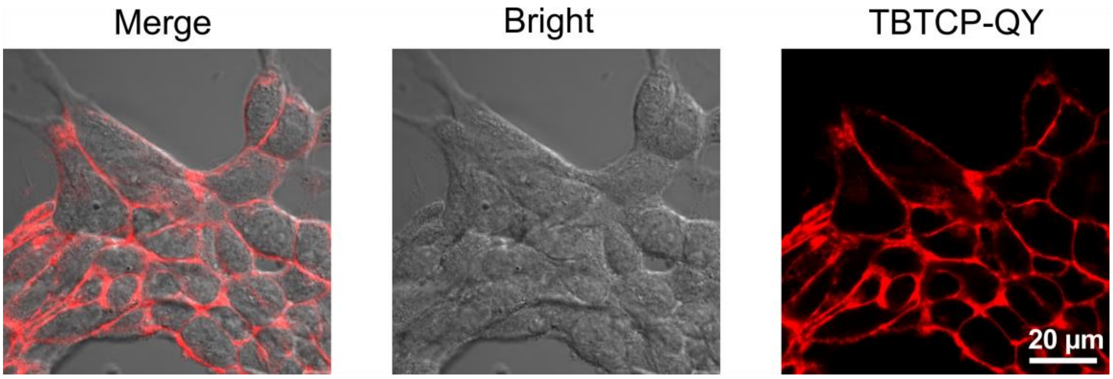


**Figure S5.** Images of the plasma membrane in HEK-293 cells stained with TBTCP-QY. The red channel used a 561 nm laser and a 570–620 nm emission filter (scale bar: 20 μm).

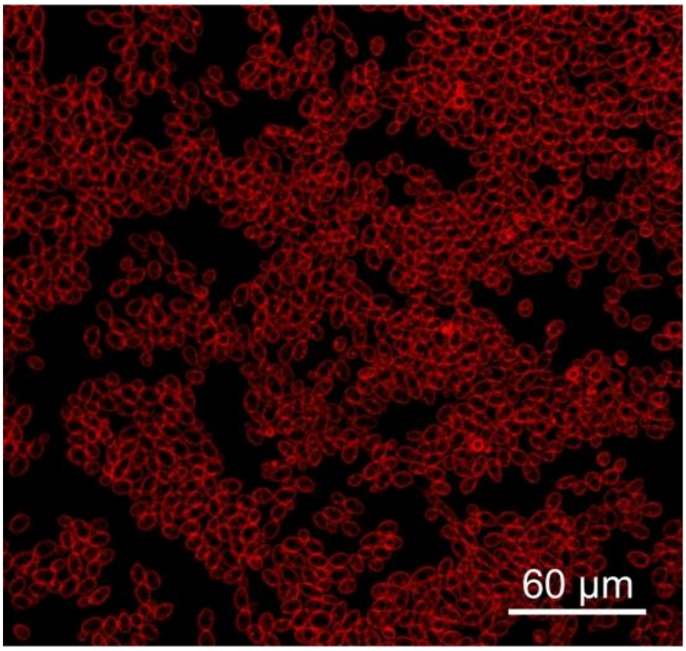


**Figure S6.** CLSM images of *C. albicans* stained with TBTCP-QY. The red channel used a 561 nm laser and a 570–620 nm emission filter (scale bar: 60 μm).

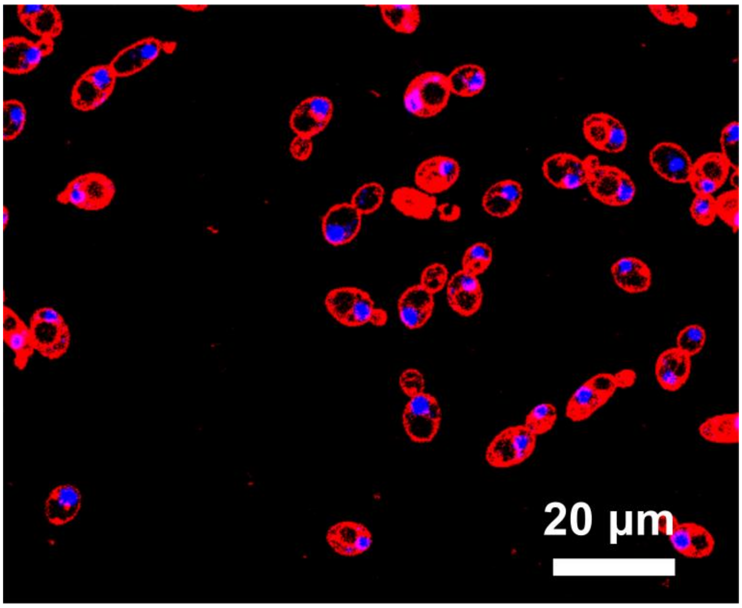


**Figure S7.** CLSM images of *C. albicans* counterstained with DAPI and TBTCP-QY. The red channel used a 561 nm laser and a 570–620 nm emission filter, and the blue channel used a 405 nm laser and a 425–475 nm emission filter (scale bar: 20 μm).

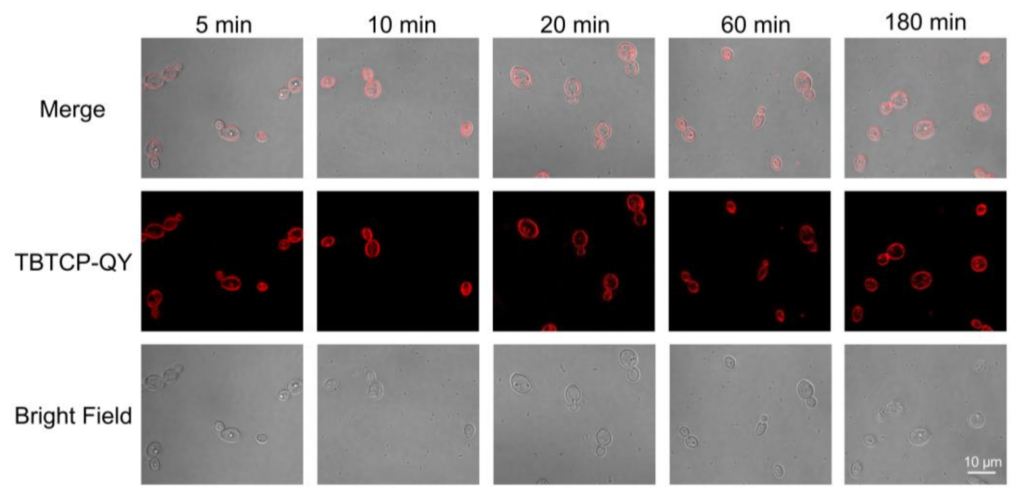


**Figure S8.** CLSM images of *C. albicans* stained with TBTCP-QY for different time. The red channel used a 561 nm laser and a 570–620 nm emission filter (scale bar: 10 μm).

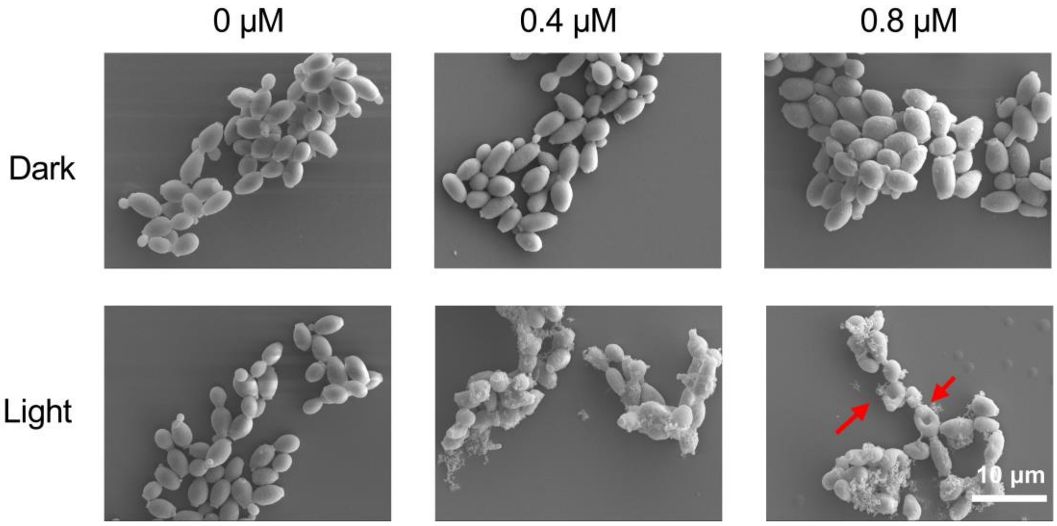


**Figure S9.** FESEM images of *C. albicans* incubated with different concentrations of TBTCP-QY with or without light irradiation for 15 min (80 mW $cm^{-2}$). The red arrows indicate deformed or broken fungal structures (scale bar: 10 μm).

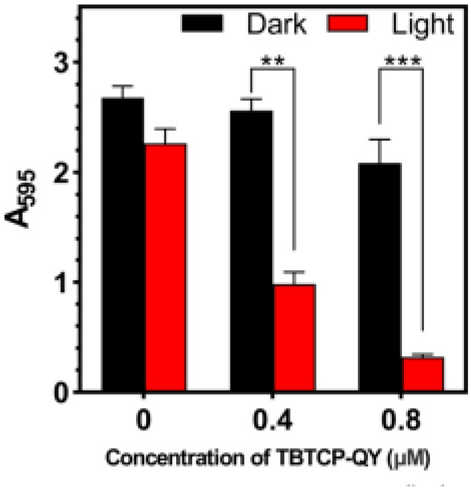


**Figure S10.** The absorbance value of *C. albicans* biofilm at 595 nm with different treatments and then stained with crystal violet. Data are expressed as the mean ± SD of 3 replicates. Statistical significance between every two groups was calculated via one-way ANOVA. * $p < 0.05$, ** $p < 0.01$, *** $p < 0.001$, **** $p < 0.0001$; ns, not significant.

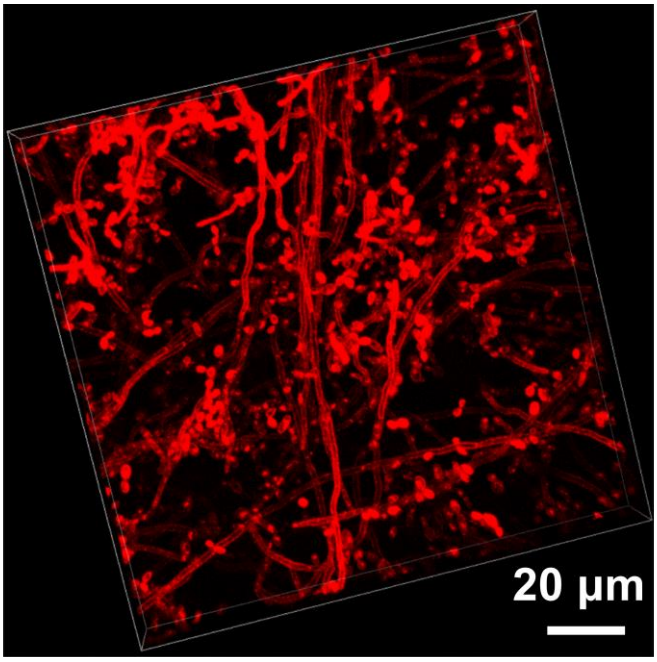


**Figure S11.** Fluorescence images of the plasma membrane of *C. albicans* in biofilms stained with TBTCP-QY. The red channel used a 561 nm laser and a 570–620 nm emission filter (scale bar: 20 μm).

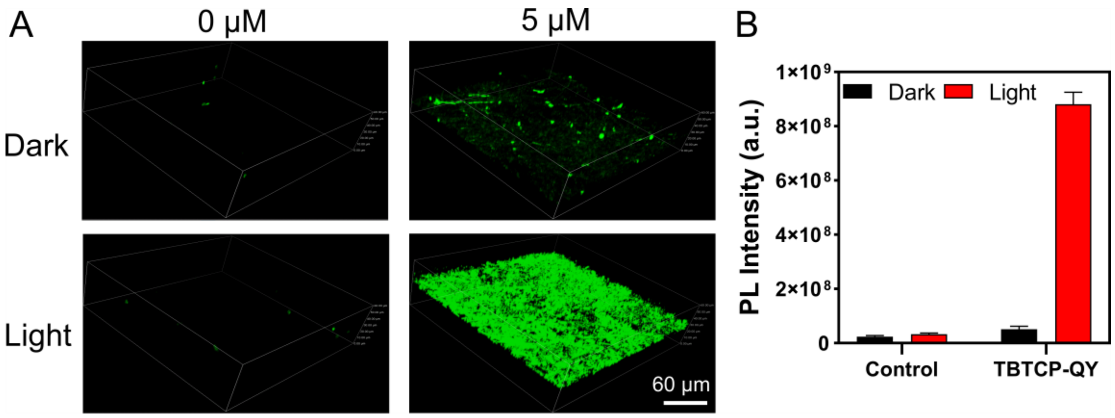


**Figure S12.** Evaluation of reactive oxygen species production in biofilms. A) CLSM 3D images of biofilms (scale bar: 60 μm) and B) quantification of the corresponding fluorescence intensity according to COMSTAT 2.0 for five random sights of *C. albicans* biofilms. Biofilms were stained with DCFH, incubated without/with 5 μM of TBTCP-QY and then irradiated with light (80 mW $cm^{-2}$). The green channel uses a 488 nm laser and a 515–550 nm emission filter. Data are shown as mean ± SD.

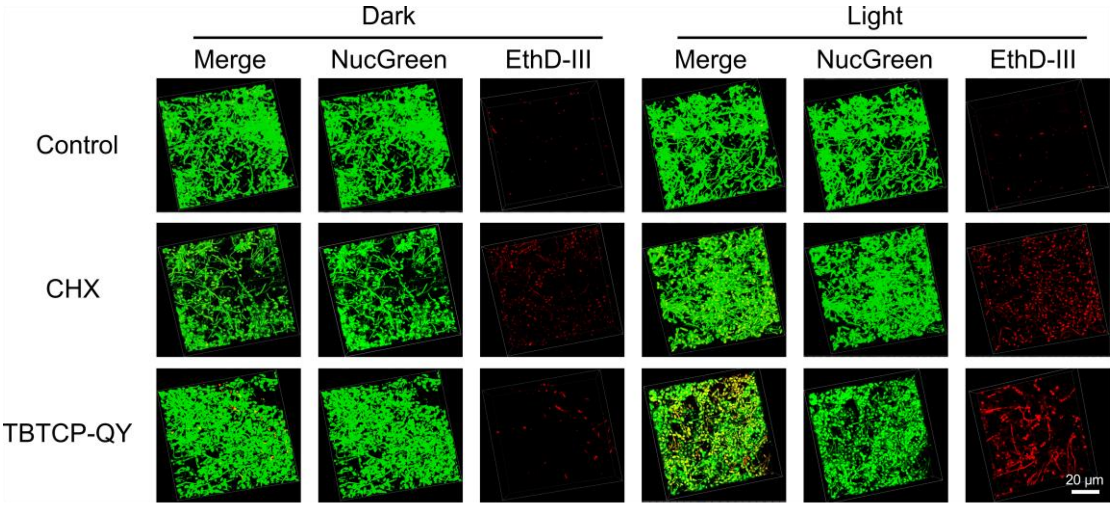


**Figure S13.** 3D images of biofilms treated with TBTCP-QY or CHX and white light irradiation and then stained with a Live & Dead$^{TM}$ activity/cytotoxicity analysis kit (UElandy). The green channel used a 488 nm laser and a 515–550 nm emission filter. The red channel used a 561 nm laser and 570–620 nm emission filter (scale bar: 20 μm).

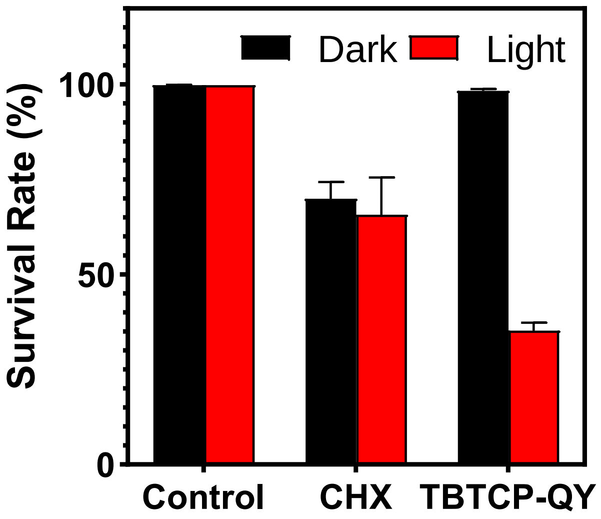


**Figure S14.** Quantification of survival rate of *C. albicans* biofilms. Data were determined from the ratio of red/green fluorescence intensity with five random regions of *C. albicans* biofilms from images of Figure 4B. Data are shown as mean ± SD.

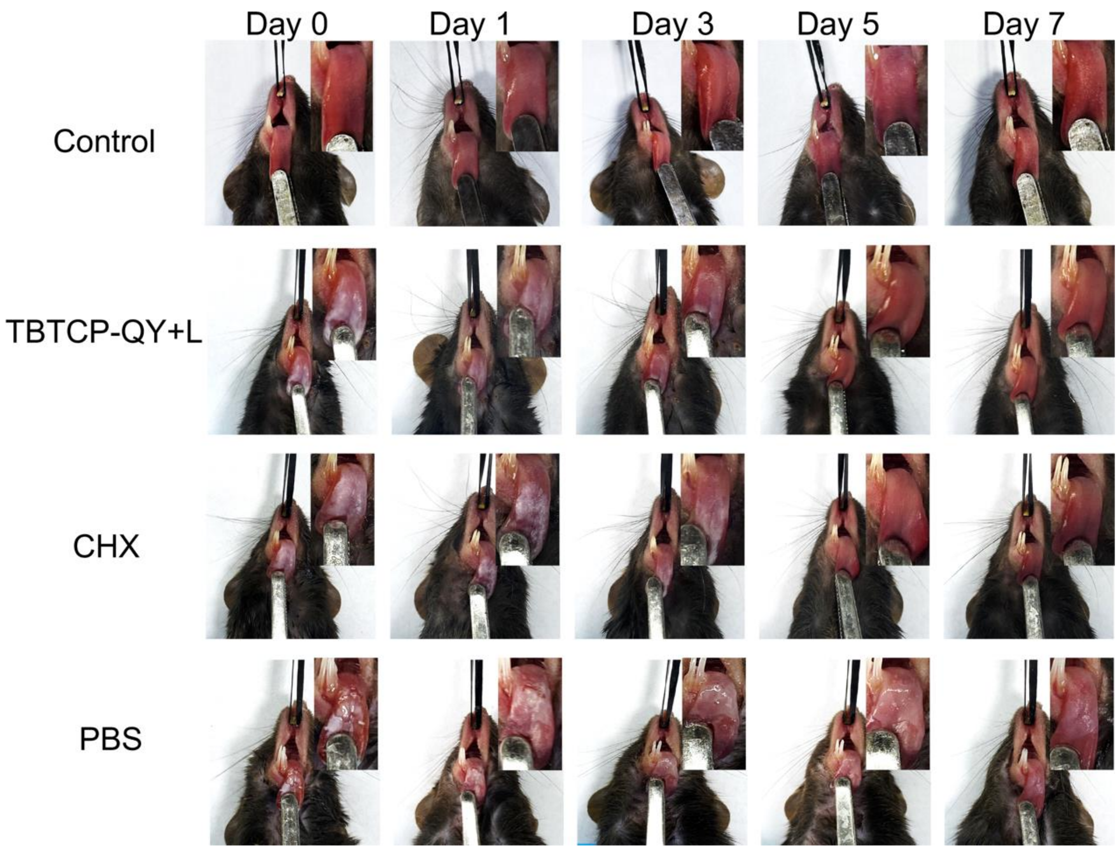


**Figure S15.** Representative images of OC mice from different treatment groups (TBTCP-QY+L, CHX and PBS) after 7 days (n = 5 mice in each group). Mice without *C. albicans* infection were used as the Control group.

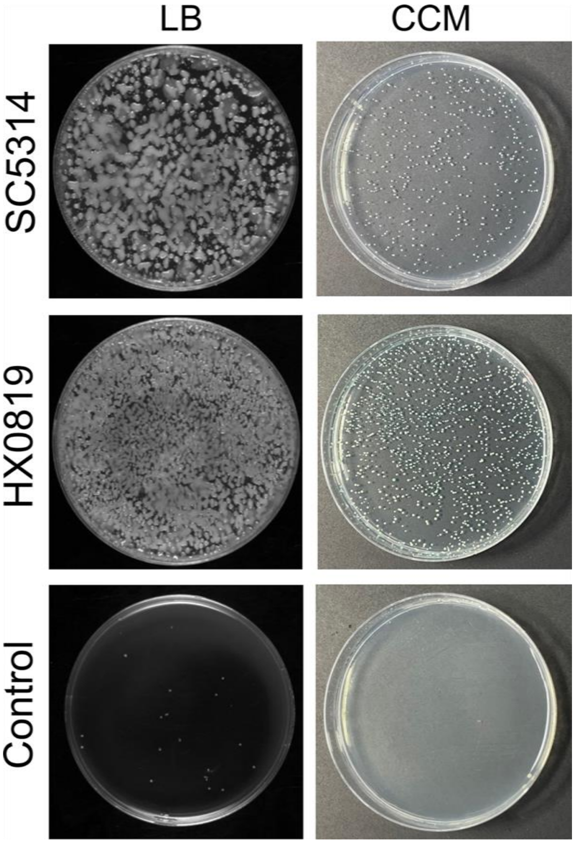


**Figure S16.** Plate images of bacteria (gray, LB agar) and fungi (green, CCM agar) from vaginal washes taken *in vivo* VVC model mice on Day 1 after treatment.

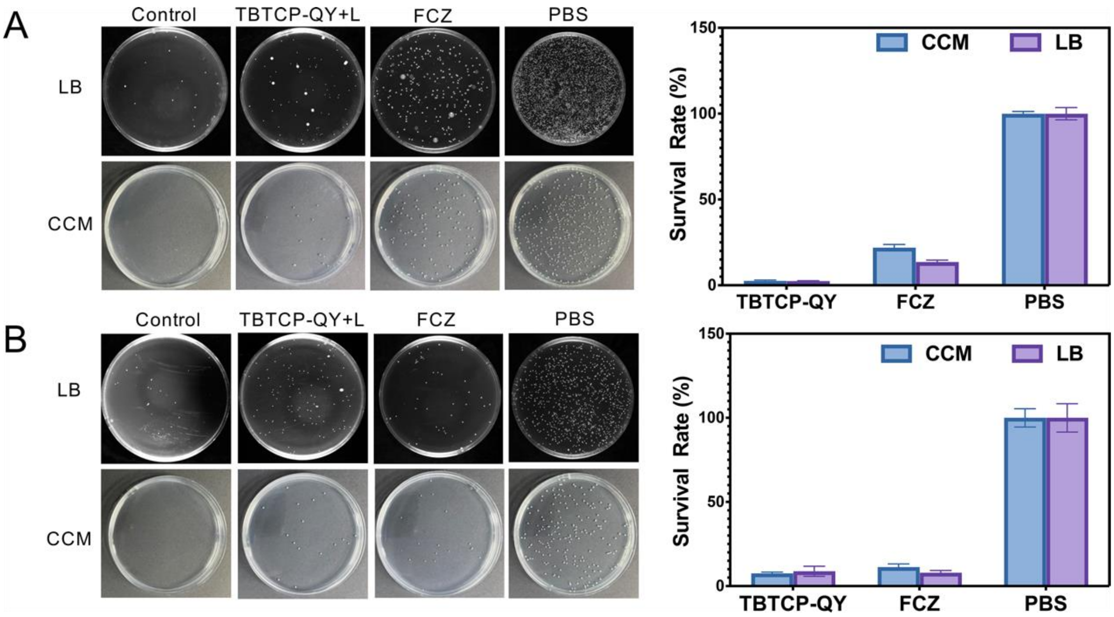


**Figure S17.** Plate images of the samples from VVC mice infected with strain A) HX0819 and strain B) SC5314 on Day 5 with different treatments. Data are shown as mean ± SD.

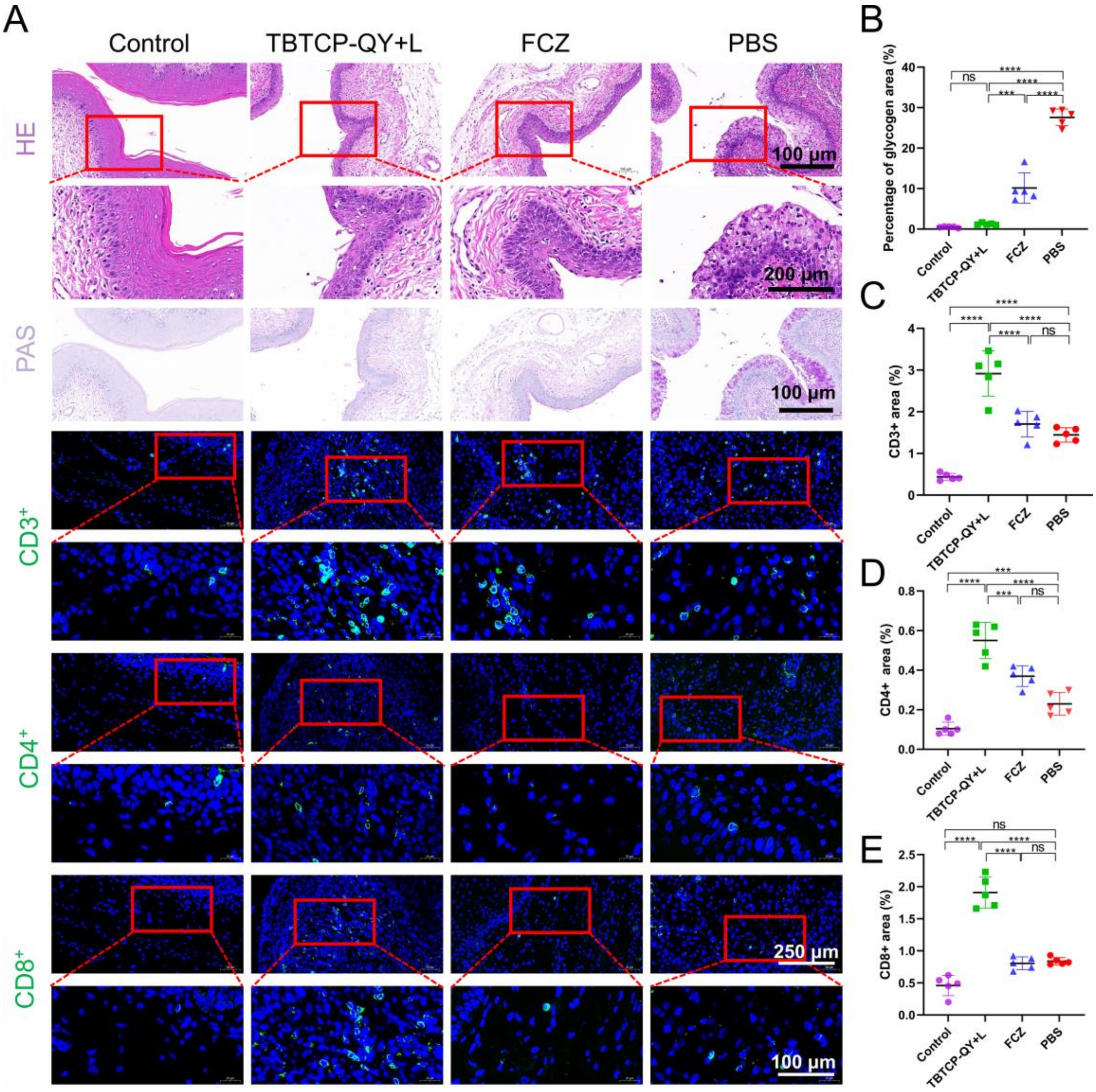


**Figure S18.** Analyses of TBTCP-QY treatment of *C. albicans* SC5314 induced VVC in mice (n = 5 mice in each group). A) H&E staining, PAS staining and expression of CD3, CD4, and CD8 in vaginal tissues from mice in different groups infected with SC5314 (TBTCP-QY+L, FCZ, PBS). Mice without *C. albicans* infection were used as controls (scale bar: 100 μm and 250 μm). B) Percentages of glycogen, C) $CD3^+$, D) $CD4^+$ and E) $CD8^+$ areas in different groups. Data are shown as mean ± SD. Statistical significance between every two groups was calculated via one-way ANOVA. * $p < 0.05$, ** $p < 0.01$, *** $p < 0.001$, **** $p < 0.0001$; ns, not significant.

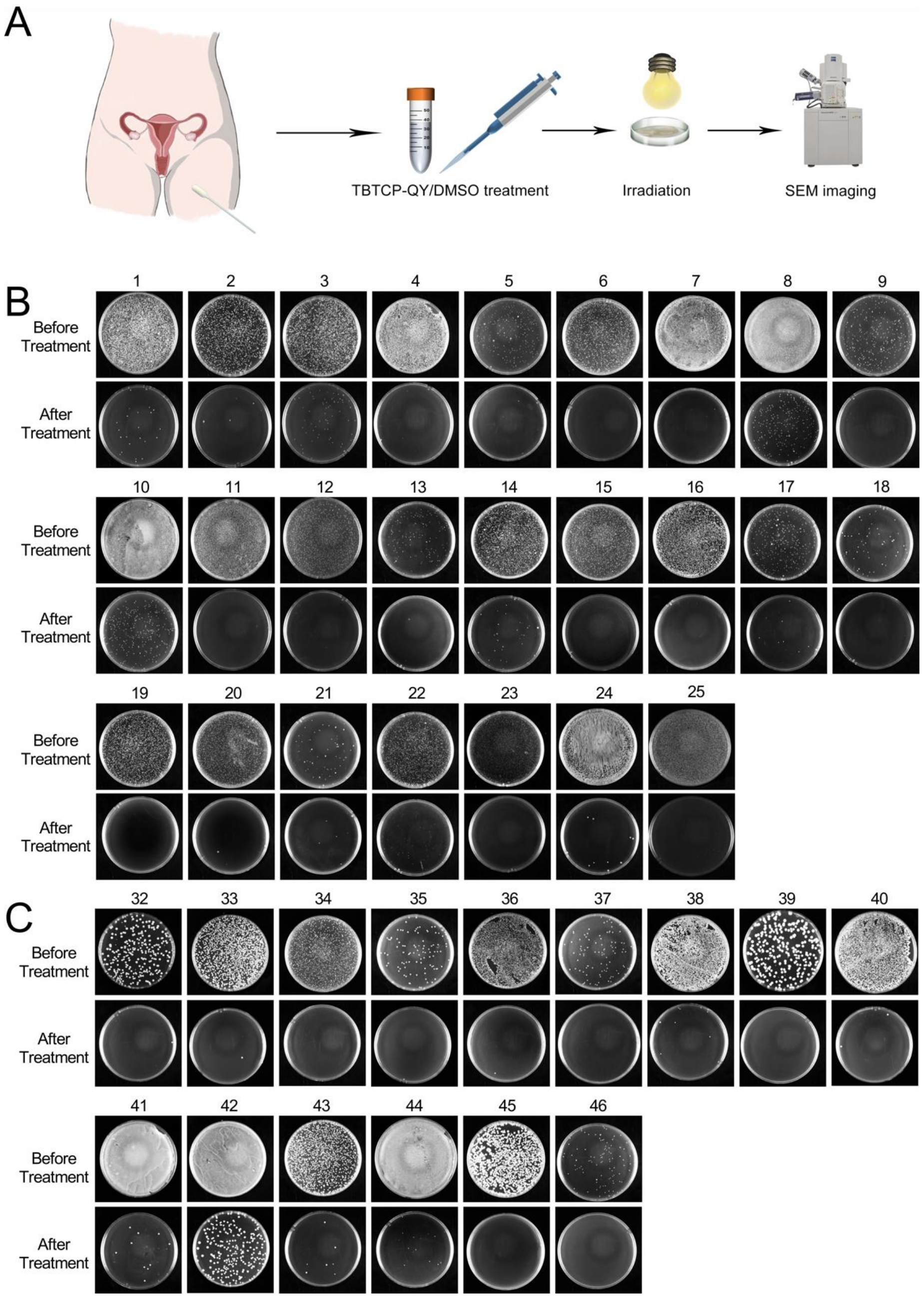


**Figure S19.** Photodynamic antifungal effect of TBTCP-QY on clinical sample. A) Schematic diagram of the experimental procedure for taking clinical samples and treating them using TBTCP-QY and white light irradiation. B**)** LB agar images of clinical samples (BV) before and after treated with TBTCP-QY and light irradiation (0.8 μM, 80 mW $cm^{-2}$). C) LB agar images of clinical samples (BV+VVC) before and after treated with TBTCP-QY and light irradiation (0.8 μM, 80 mW $cm^{-2}$).

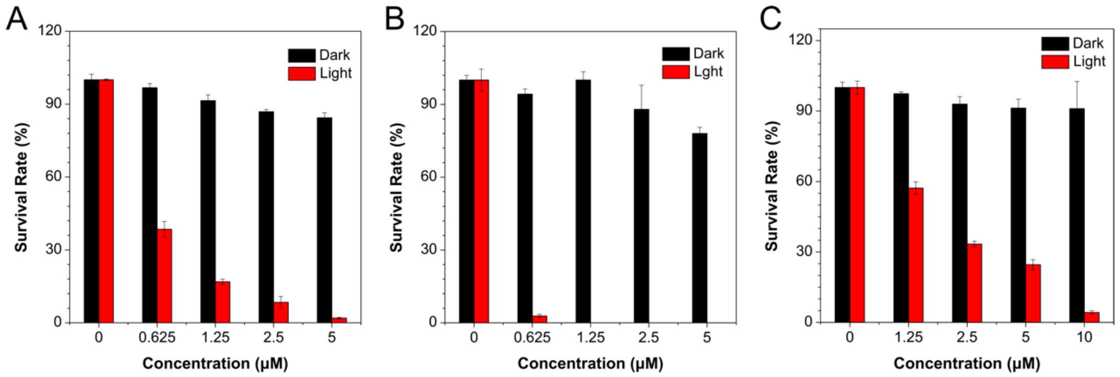


**Figure S20.** Survival rate A) *S. aureus*, B) MRSA and C) *E. coli* after treatment of TBTCP-QY, evaluated by serial dilution test on LB agar. Bacteria were treated without/with varied concentrations of TBTCP-QY, followed by storage in dark or white light irradiation (20 mW cm$^{-2}$) for 30 min. Data are presented as mean ± SD with at least 3 replicates.

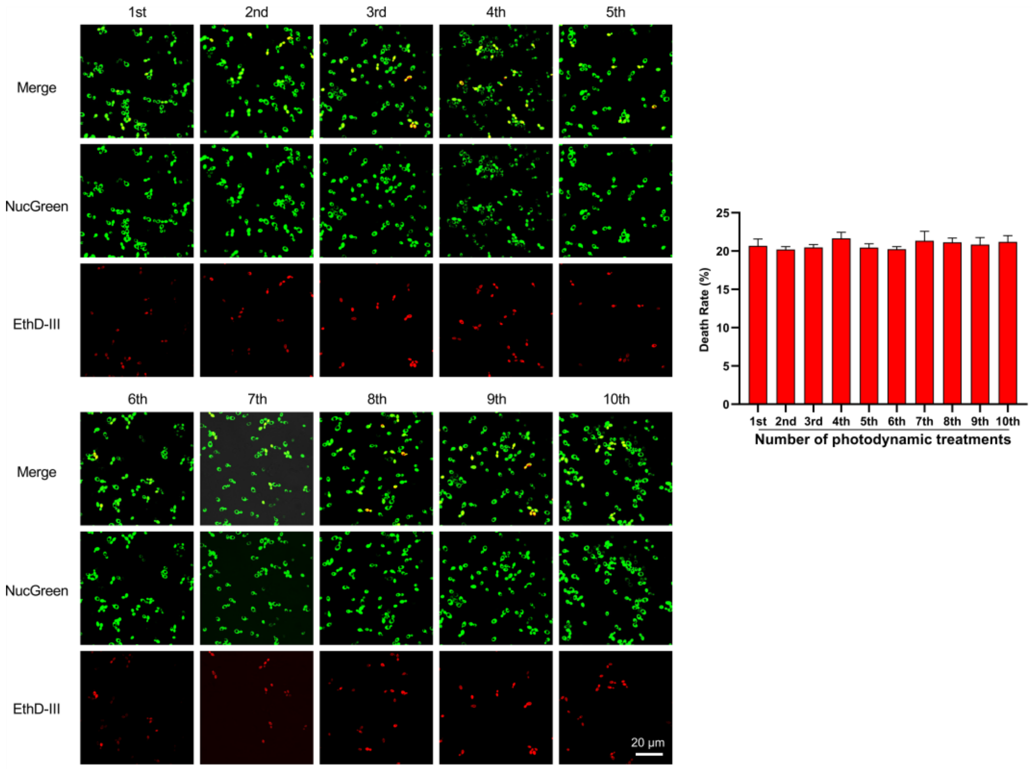


**Figure S21.** PDI of *C. albicans* HX0819 after ten consecutive cycles treatment with 0.3 µM TBTCP-QY for 15 min and irradiation with white light for 15 min (80 mW cm$^{-2}$) followed by staining with a Live & Dead$^{TM}$ activity/cytotoxicity analysis kit (UElandy). The green channel used a 488 nm laser and a 515–550 nm emission filter, and the red channel used a 561 nm laser and 570–620 nm emission filter (scale bar: 20 µm).

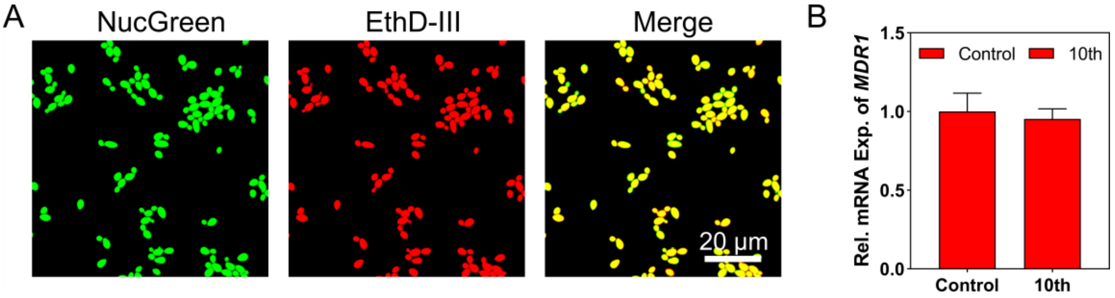


**Figure S22.** Photodynamic antimicrobial effect of TBTCP-QY on drug resistant *C. albicans* HX0819. A) Photodynamic inactivation of *C. albicans* HX0819 after ten consecutive cycles of treatment with 0.8 μM TBTCP-QY for 15 min and irradiation with white light for 15 min (80 mW $cm^{-2}$) followed by staining with a Live & Dead$^{TM}$ activity/cytotoxicity analysis kit (Everbright, USA). The green channel used a 488 nm laser and a 515–550 nm emission filter, and the red channel used a 561 nm laser and 570–620 nm emission filter (scale bar: 20 μm). B) mRNA expression of *MDR1* before and after *C. albicans* HX0819 was treated with ten PDI cycles. Untreated fungi were used as control. Data are shown as mean ± SD.

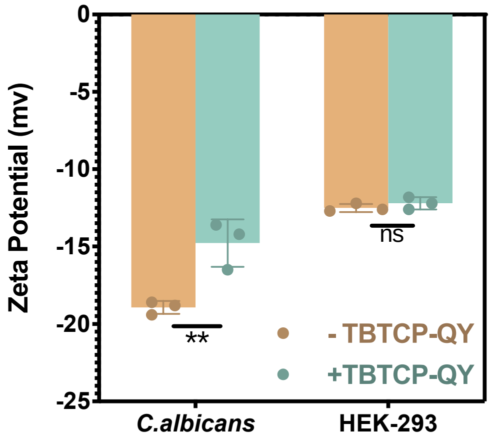


**Figure S23.** Zeta potential results of *C. albicans* and HEK-293 cells in PBS pretreated with or without 5 μM TBTCP-QY. Data are shown as mean ± SD. Statistical significance between every two groups was calculated via one-way ANOVA. * $p < 0.05$, ** $p < 0.01$, *** $p < 0.001$, **** $p < 0.0001$; ns, not significant.

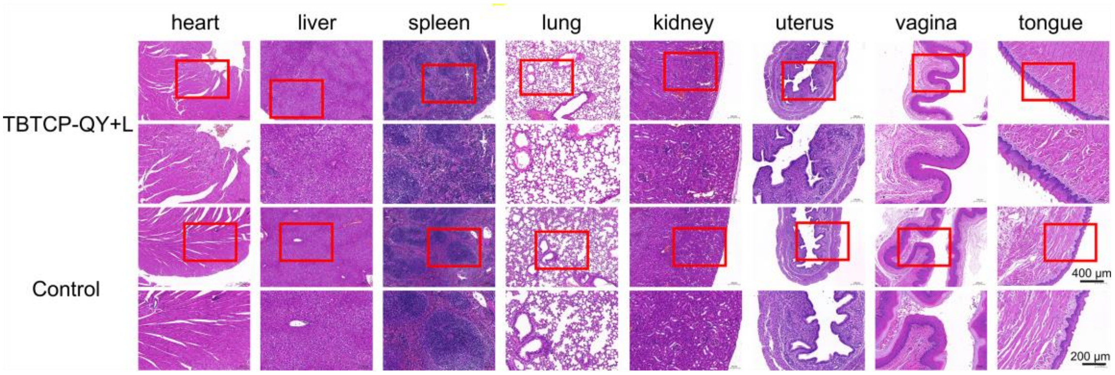


**Figure S24.** H&E staining images of mouse organs after 10 days of treatment with TBTCP-QY (n = 5 mice in each group, scale bar: 200 μm and 400 μm).

**Table S1.** Primers used in qRT–PCR for detecting *GPD1*, *ALS3*, *EAP1*, *HWP1*, *MDR1*, *SAP1*, and *SAP2* levels.

| **qRT-PCR primers** | **Fp (5'to 3')** | **Rp (5'to 3')** |
|---|---|---|
| *GPD1* | AGTATGTGGAGCTTTACTGGGA | CAGAAACACCAGCAACATCTTC |
| *ALS3* | CTGGACCACCAGGAAACACT | ACCTGGAGGAGCAGTGAAAG |
| *EAP1* | TGTGATGGCGGTTCTTGTTC | GGTAGTGACGGTGATGATAGTGACA |
| *HWP1* | CGGAATCTAGTGCTGTCGTCTCT | CGACACTTGAGTAATTGGCAGATG |
| *MDR1* | AGTTGCTTGGGGTAGTTCCG | TGCTCTCAACTTTGGTCCGT |
| *SAP1* | GAACCAAGGAGTTATTGCCAAGA | TTTGTCCAGTGGCAGCATTG |
| *SAP2* | GTCACTTTAAAAAAACAAGGAGTCATTG | TATTTGTCCCGTGGCAGCAT |

**Table S2.** Absolute expression of immune factors in mice.

| Name | TBTCP-QY+L | PBS | CHX |
|---|---|---|---|
| GCSF | 39.21 | 2620.21 | 1045.16 |
| GM-CSF | 62.17 | 83.81 | 83.91 |
| IL-1 alpha | 1118.25 | 134.93 | 603.90 |
| IL-1 beta | 105.44 | 112.05 | 108.34 |
| IL-2 | 50.87 | 82.17 | 80.70 |
| IL3 | 29.67 | 38.91 | 67.37 |
| IL-4 | 139.09 | 130.50 | 245.80 |
| IL-5 | 87.16 | 91.98 | 124.63 |
| IL-6 | 116.57 | 914.65 | 1705.33 |
| IL-7 | 87.96 | 89.33 | 107.60 |
| IL-9 | 254.59 | 199.83 | 200.39 |
| IL-10 | 177.76 | 170.42 | 429.91 |
| IL-12 p70 | 167.43 | 170.88 | 476.55 |
| IL-13 | 19.78 | 19.61 | 68.61 |
| IL-15 | 27.02 | 63.81 | 227.05 |
| IL-17A | 66.50 | 69.80 | 136.23 |
| IL-21 | 134.58 | 105.52 | 172.26 |
| IL-23 | 5.30 | 0.93 | 27.15 |
| INF-gamma | 306.78 | 255.62 | 394.62 |
| TNF-alpha | 103.85 | 96.41 | 138.70 |

**Table S3.** Patients clinical sample information.

| Sample | Age | Whether had given birth | Whether in gestation period | Whether had a history of vaginitis | Types of vaginitis |
|---|---|---|---|---|---|
| **1** | 31 | √ | × | √ | BV |
| **2** | 25 | × | × | × | BV |
| **3** | 34 | √ | × | √ | BV |
| **4** | 33 | √ | × | × | BV |
| **5** | 33 | √ | × | √ | BV |
| **6** | 19 | × | × | × | BV |
| **7** | 46 | √ | × | √ | BV |
| **8** | 28 | √ | √ | × | BV |
| **9** | 33 | √ | × | √ | BV |
| **10** | 38 | √ | × | × | BV |
| **11** | 52 | √ | × | × | BV |
| **12** | 25 | × | × | √ | BV |
| **13** | 30 | √ | × | × | BV |
| **14** | 46 | √ | × | √ | BV |
| **15** | 35 | √ | × | √ | BV |
| **16** | 36 | √ | × | √ | BV |
| **17** | 50 | √ | × | × | BV |
| **18** | 36 | √ | × | × | BV |
| **19** | 48 | √ | × | × | BV |
| **20** | 43 | √ | × | √ | BV |
| **21** | 49 | √ | × | × | BV |
| **22** | 45 | √ | × | × | BV |
| **23** | 67 | √ | × | × | BV |
| **24** | 50 | √ | × | × | BV |
| **25** | 31 | √ | × | × | BV |

| | | | | | |
|---|---|---|---|---|---|
| **26** | 36 | √ | × | √ | VVC |
| **27** | 39 | √ | × | √ | VVC |
| **28** | 29 | √ | × | √ | VVC |
| **29** | 41 | √ | × | √ | VVC |
| **30** | 45 | √ | × | √ | VVC |
| **31** | 31 | √ | √ | √ | VVC |
| **32** | 42 | √ | × | √ | BV+VVC |
| **33** | 32 | √ | × | √ | BV+VVC |
| **34** | 34 | √ | × | × | BV+VVC |
| **35** | 39 | √ | × | × | BV+VVC |
| **36** | 32 | √ | × | × | BV+VVC |
| **37** | 38 | × | × | × | BV+VVC |
| **38** | 33 | √ | × | √ | BV+VVC |
| **39** | 24 | × | × | √ | BV+VVC |
| **40** | 48 | √ | × | √ | BV+VVC |
| **41** | 25 | √ | × | × | BV+VVC |
| **42** | 52 | √ | × | √ | BV+VVC |
| **43** | 27 | √ | × | √ | BV+VVC |
| **44** | 24 | √ | × | √ | BV+VVC |
| **45** | 25 | √ | × | √ | BV+VVC |
| **46** | 38 | √ | × | √ | BV+VVC |

**Table S4.** Summary of patients' information.

| Characteristics of patients | All patients (n = 46) |
|---|---|
| **Median age (range) - yr** | 36 (19–67) |
| **Type** | |
| **VVC** | 6 (13.04%) |
| **BV** | 25 (43.35%) |
| **BV+VVC** | 15 (32.61%) |

**Table S5.** Zeta potential results of *C. albicans* and HEK-293 cells in PBS solution pretreated with or without 5 μM TBTCP-QY.

| Group | *C. albicans* | | HEK-293 | |
|---|---|---|---|---|
| | Without TBTCP-QY | With TBTCP-QY | Without TBTCP-QY | With TBTCP-QY |
| ζ (mV) | - 18.93 ± 0.42 | - 14.77 ± 1.53 | - 12.5 ± 0.26 | - 12.2 ± 0.4 |

**Table S6.** Antibodies used in this study.

| Antibodies | Company | Description | Catalog Number | Dilution |
|---|---|---|---|---|
| α-SMA | BOSTER | Mouse monoclonal antibody | BM0002 | 1:200 |
| CD31 | ABCAM | Rabbit monoclonal antibody | ab182981 | 1:1000 |
| iNOS | PTG | Rabbit polyclonal antibody | 18985-1-AP | 1:500 |
| MMR | Servicebio | Rabbit polyclonal antibody | GB113497 | 1:500 |
| MPO | ABCAM | Rabbit monoclonal antibody | ab208670 | 1:500 |
| CD3 | ABCAM | Rabbit monoclonal antibody | ab237721 | 1:1000 |
| CD4 | ABCAM | Rabbit monoclonal antibody | ab183685 | 1:500 |
| CD8 | ABCAM | Rabbit monoclonal antibody | ab217344 | 1:500 |
| Secondary antibody | Jackon | CY3-conjugated goat anti-rabbit IgG | 111-165-003 | 1:500 |
| Secondary antibody | Jackon | Alexa Fluor® 488-conjugated goat anti-rabbit IgG | 111-545-003 | 1:200 |
| Secondary antibody for α-SMA | DAKO | HRP goat anti-rabbit IgG | K5007 | 1:4000 |
